# Switching azide and alkyne tags on bioorthogonal reporters in metabolic labeling of sialylated glycoconjugates: a comparative study

**DOI:** 10.1038/s41598-022-26521-3

**Published:** 2022-12-22

**Authors:** Jodie Scache, Vincent Rigolot, Cédric Lion, Marlène Mortuaire, Tony Lefebvre, Christophe Biot, Anne-Sophie Vercoutter-Edouart

**Affiliations:** Université de Lille, CNRS, UMR 8576, UGSF, Unité de Glycobiologie Structurale et Fonctionnelle, 59000 Lille, France

**Keywords:** Glycobiology, Cellular imaging

## Abstract

Sialylation of cell surface glycans plays an essential role in cell–cell interaction and communication of cells with their microenvironment. Among the tools that have been developed for the study of sialylation in living cells, metabolic oligosaccharide engineering (MOE) exploits the biosynthetic pathway of sialic acid (Sia) to incorporate unnatural monosaccharides into nascent sialylatedglycoconjugates, followed by their detection by a bioorthogonal ligation of a molecular probe. Among bioorthogonal reactions, the copper-catalyzed azide-alkyne cycloaddition (CuAAC) is the only ligation where both reactive tags can be switched on the chemical reporter or on the probe, making this reaction very flexible and adaptable to various labeling strategies. Azide- and alkyne-modified ManNAc and Sia reporters have been widely used, but per-*O*-acetylated ManNAz (Ac_4_ManNAz) remains the most popular choice so far for tracking intracellular processing of sialoglycans and cell surface sialylation in various cells. Taking advantage of CuAAC, we compared the metabolic incorporation of ManNAl, ManNAz, SiaNAl, SiaNAz and Ac_4_ManNAz in the human colon cell lines CCD841CoN, HT29 and HCT116, and in the two gold standard cell lines, HEK293 and HeLa. Using complementary approaches, we showed marked differences in the efficiency of labeling of sialoglycoproteins between the different chemical reporters in a given cell line, and that switching the azide and alkyne bioorthogonal tags on the analogs highly impacted their metabolic incorporation in the human colon cell lines. Our results also indicated that ManNAz was the most promiscuous metabolized reporter to study sialylation in these cells.

## Introduction

The N-Acetyl neuraminic acid (Neu5Ac, Sia) is the most abundant member of the sialic acid family. This negatively charged monosaccharide is located at the non-reductive end of glycan chains carried by glycoproteins and glycosphingolipids at the surface of human cells. Sialylation plays an important role in cell–cell recognition and cell-microenvironment interactions in physiological or pathological context. For example, changes of sialylation are associated with cancer progression and metastasis, especially in colon cancer^1,2^, and sialylated cancer-associated antigens, such as sialyl-Tn, constitute targets for therapeutic strategies^3,4^. Yet, identification of proteins whose sialylation is impaired is still of interest to better understand the molecular and cellular mechanisms that are affected during tumor progression, such as migration and invasion properties, and immune escape mechanisms^5^.

**Figure 1:**
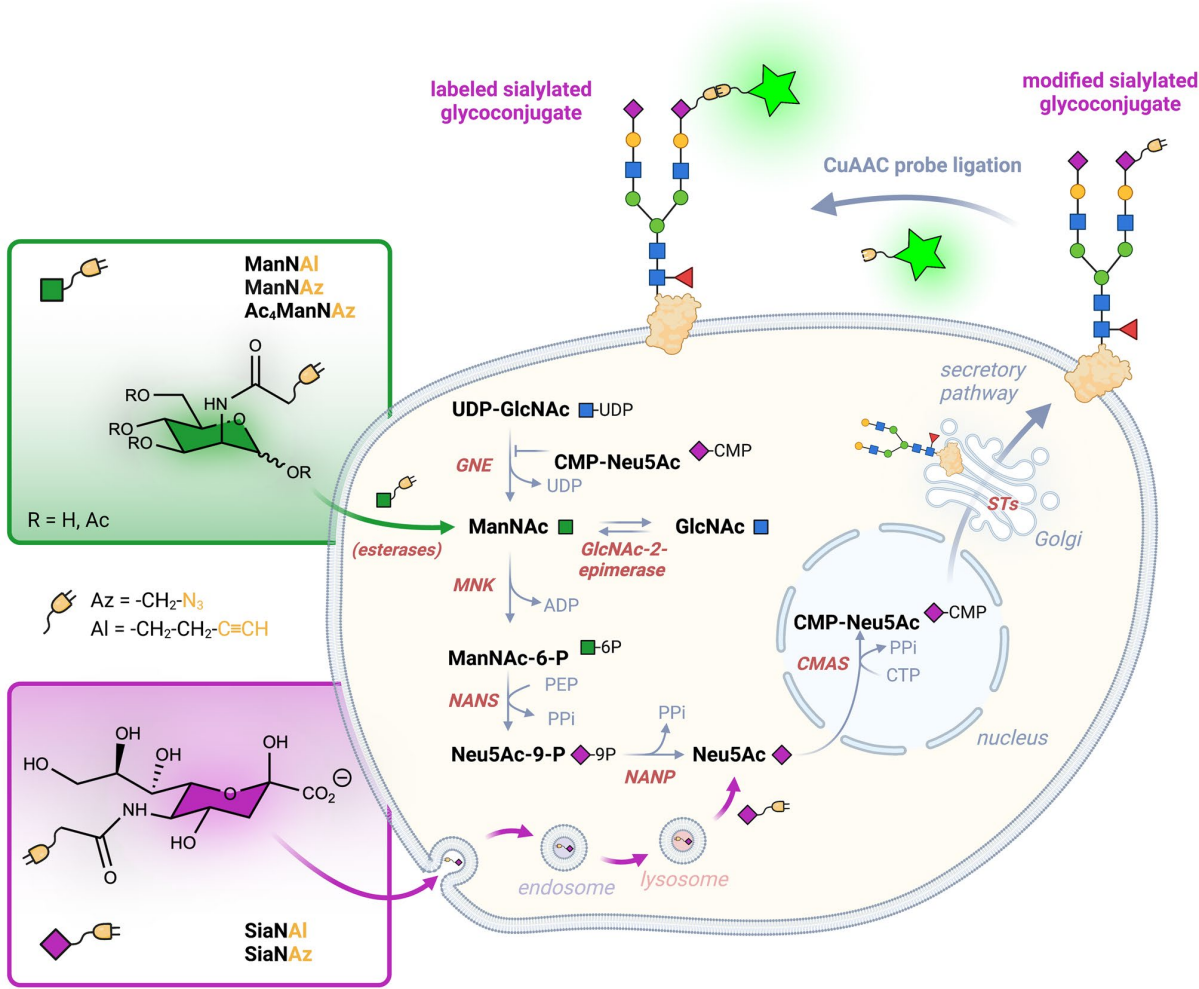
Metabolic incorporation of ManNAc and nonnatural ManNAc and Sia (Neu5Ac) analogs in the sialic acid biosynthetic pathway. ManNAc is synthesized from UDP-GlcNAc and GlcNAc by UDP-GlcNAc 2-epimerase (GNE) and GlcNAc-2-epimerase (RENBP), respectively. ManNAc and ManNAc analogs are phosphorylated by ManNAc 6-kinase (MNK) to yield ManNAc-6-phosphate (ManNAc-6-P). GNE/MNK is a bifunctional enzyme whose GNE activity is inhibited by the final product CMP-NeuAc. The condensation of ManNAc-6-P with phosphoenolpyruvate (PEP) is catalyzed by *N*-acetylneuraminate synthase (NANS) to produce *N*-acetyl neuraminic acid-9-phosphate (Neu5Ac-9-P), which is then dephosphorylated by sialic acid phosphatase (NANP) to yield Neu5Ac. Cytosolic pool of Neu5Ac can also be provided by the salvage pathway through endocytosis of free sialic acid and lysosomal recycling. Transport of Neu5Ac to the nucleus enables CMP-sialic acid synthetase (CMAS) to produce CMP-NeuAc. After its transport into the Golgi apparatus, CMP-NeuAc is utilized by the sialyltransferases (STs) for the terminal glycosylation of glycoconjugates that are ultimately secreted or destined for the cell surface or organelles, as illustrated here with a transmembrane *N*-glycoprotein.

During the last decade, huge efforts have been made to develop novel chemical reporters for the biocompatible labeling of sialylated glycoconjugates (GCs) using metabolic oligosaccharide engineering (MOE). Such a strategy exploits the biosynthetic pathway of sialic acid to incorporate unnatural Sia derivatives into GCs in living cells^6,7^. Sia or ManNAc analogs can both be used as two different entry points into the metabolic route (Fig. 1). Synthetic ManNAc analogs are incorporated into the de novo biosynthetic pathway thanks to the *N*-acetylmannosamine kinase (MNK) activity of the bifunctional enzyme GNE (UDP-GlcNAc 2-epimerase). In contrast, GNE is not required for Sia analogs which go through the endo-lysosomal recycling pathway before being exported into the cytosol via SLC17A5 (Fig. 1). Once synthetized in the nucleus by CMP-sialic acid synthetase (CMAS), the functionalized CMP-Sia is then transported into the Golgi where it is used by sialyltransferases (Fig. 1). Among the reactive chemical groups, alkynes and azides are totally absent from living organisms, small, relatively stable and chemically inert in the cellular environment. This makes the metabolic chemical reporters bearing them structurally as close as possible to the natural analogs, offering great confidence in their physiological behaviors as a model. Modified alkyne- or azide ManNAc and Sia monosaccharides are usually used at high concentrations (> 0.5–1 mM) because of their high polarity that limits their cellular uptake^8–10^. For that reason, per-*O*-acetylated analogs are preferentially used, due to their hydrophobic properties that facilitate their passive diffusion across the cell membrane, allowing lower working concentrations (20–100 µM)^7,11^. Both peracetylated alkyne and azide-modified ManNAc and Sia can be used. However, differences in the efficiency of incorporation and cell surface labelling were reported, as shown for example for Ac_4_ManNAz and Ac_4_ManNAl^12,13^, or Ac_5_SiaNAz and Ac_5_SiaNAl (referred to as Ac_5_SiaNPoc)^14,15^. Sia reporters, whose synthesis is demanding, seem to be more efficiently incorporated into sialylated GCs compared with their ManNAc analogs^9,14^. Indeed, Sia analogs enter cells via the salvage pathway and are directly available for CMP activation in the nucleus, while the conversion of ManNAc analogs into the modified nucleotide-sugar requires three additional enzymatic steps (Fig. 1)^7^. For this reason, MOE using ManNAc and Sia analogs is useful to study defects in the sialic acid biosynthetic and recycling pathways^8,9,16^.

Among the bioorthogonal ligations used to label the modified sialylated GCs with a fluorescent or biotinylated probe, cycloadditions including the Copper-catalyzed azide-alkyne cycloaddition (CuAAC) and the strain-promoted [3 + 2] alkyne-azide cycloaddition (SPAAC), have been widely used in vitro and in vivo^7–9,12,13,17–22^. CuAAC is the only bioorthogonal reaction in which alkynes and azides can be introduced indifferently either on the metabolic analog or on the probe for the ligation, making this reaction very flexible and adaptable to various labeling strategies. In contrast, only azides are adapted for a bioorthogonal ligation via SPAAC reaction, which is easier to apply in living systems than CuAAC due to the absence of copper associated cytotoxicity, but generally suffers from poorer kinetics and specificity especially in the intracellular environment^23^. Albeit numerous works using MOE to label sialylated glycans have been reported, the comparison of alkyne- or azide-functionalized ManNAc and Sia free monosaccharides has not been done so far. Therefore, in this work, we utilized the advantage of CuAAC to swap the azide and alkyne tags between the unnatural monosaccharides and the probes. We compared the metabolic incorporation efficiency of ManNAl, ManNAz, SiaNAl, SiaNAz and peracetylated Ac_4_ManNAz in several human cell lines (Fig. S1). We chose the two widely used HEK293 and HeLa cells, and the three human colon cell lines CCD841CoN, HT29 and HCT116. The latter are particularly interesting as in vitro cellular models of colon cancer progression for studying how cytosolic *O*-GlcNAcylation can interfere with glycosylation that occurs in the secretory pathway^24,25^. After ligation with the appropriate probe, the labeling of sialylated GCs was compared by flow cytometry, Western blotting and confocal fluorescent microscopy. We showed marked differences in the efficiency of cell surface glycan labeling between the five different bioorthogonal reporters in a given cell line. Moreover, although Ac_4_ManNAz has been extensively used in many cell types, we found that ManNAz appears to be the bioorthogonal reporter of choice to detect sialylated GCs at the cell surface of human colon cells.

## Results

### Comparison of labeling of cell surface sialoglyconjugates by flow cytometry

Monosaccharides are polar molecules that are unable to cross the plasma membrane by passive diffusion. Therefore, the usual strategy uses peracetylated analogs, which are capable of passive diffusion and are then hydrolyzed in the cytosol by non-specific esterases to regenerate the free sugar. Unlike some other monosaccharides studied in metabolic glycoengineering studies, ManNAc and its derivatives are capable of crossing the plasma membrane in their free-hydroxyl form by an active transport mechanism that is yet unidentified^9^, therefore unprotected ManNAc derivatives have also been used to label sialoglycoconjugates. In agreement with previous studies, we used 500 µM of the free-hydroxyl analogs^9,10^. Ac_4_ManNAz is usually used in a range of 20 to 100 µM^7,11^. However, we found that 100 µM Ac_4_ManNAz reduced approximately by 40% the cellular growth of CCD841CoN, HT29 and HCT116 human colon cell lines (Fig. S2), as reported in another cell lines^26^. Therefore, the experiments were carried out with 50 µM Ac_4_ManNAz. Moreover, high concentrations of Ac_4_ManNAz can lead to S-glyco-modification side-reactions between peracetylated monosaccharides and protein cysteine residues, inducing nonspecific labeling of proteins that cannot occur with non-peracetylated analogs^27,28^. Western blot was used to check the expression of GNE, the first enzyme required to metabolize the unnatural ManNAc analogs into the sialic acid biosynthesis pathway^29^ (Fig. 1). Our result showed that GNE was expressed at a similar level in the different cell lines (Fig. S3). However, HT29 cells expressed two isoforms of GNE, GNE1 (lower band, ~ 79 kDa) and GNE2 (higher band, ~ 82 kDa) at the same level, while only GNE1 (later referred to as GNE) was strongly detected in the other cell lines^30^. Incubation of cells with ManNAc or ManNAz did not change the steady-state level of any of the two GNE isoforms (Fig. S3).

**Figure 2:**
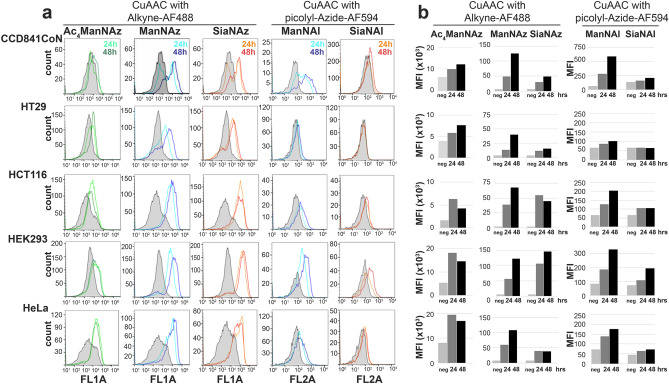
Flow cytometry analysis of labeled sialoglyconjugates at cell surface. Cells were incubated 24 h (in light color) or 48 h (in dark color) with the analogs before CuAAC using the appropriate fluorescent probe. For the negative controls (in grey), CuAAC was performed on cells cultured with ManNAc. (**a**) Flow cytometry histograms and (**b**) histograms showing the mean of fluorescent intensity (MFI). Results are representative of three experiments.

Flow cytometry analysis was performed to measure cell surface fluorescence after 24 h and 48 h of metabolic incorporation with each analog (Fig. 2). It should be noted that chelation-assisted CuAAC was carried out for the detection of cell surface alkyne-modified GCs by flow cytometry. Picolyl-azide probes (here picolyl-azide-AF594) contain an internal copper-chelating moiety that reacts faster and in a more robust manner with alkyne-modified biomolecules compared with non-chelating azide probes^21,31^. In order to point out the marked differences in MFIs, histograms are presented with raw means of fluorescence intensity (MFIs) rather than relative MFIs (Fig. 2b). This is illustrated when comparing Ac_4_ManNAz and ManNAz for example: the cell surface fluorescence increased from ten-fold to 40-fold in cells cultured with ManNAz compared with the negative controls, whereas it only increased from twice to three-fold with Ac_4_ManNAz (Fig. 2b). Relative MFIs and statistical analysis are shown in Fig. S4. Whatever the analog and the cell line, the differences of MFI observed between 24 and 48 h of incubation were not significant, except for ManNAl in CCD841CoN, HCT116 and HEK293 cells (Figs. 2, S4). In these cells, there was an increase of almost two times of cell surface fluorescence between 24 and 48 h of incorporation of ManNAl. Kinetics of incorporation and increase of fluorescence intensity were similar between ManNAz and ManNAl, except in HT29 cells which did not metabolize ManNAl (Fig. 2).

When comparing the metabolic incorporation of SiaNAz and SiaNAl to their ManNAc counterparts, we noted that the relative MFI obtained with SiaNAz was as strong as ManNAz in HCT116, HEK293 and HeLa cells (Fig. S4). In contrast, the SiaNAz-induced relative cell surface fluorescence was reduced by about 60% in CCD841CoN and HT29 cells compared to ManNAz (Fig. S4). The reduced metabolic conversion of Sia compared to ManNAc chemical reporters was even more obvious with the alkyne tag: Relative MFIs were 50–60% lower than the ones obtained with ManNAl in all the cell lines except HT29 cells which did not metabolize SiaNAl at all (Figs. 2, S4).

### Comparison of in cell glycoconjugates labeling by confocal microscopy

**Figure 3:**
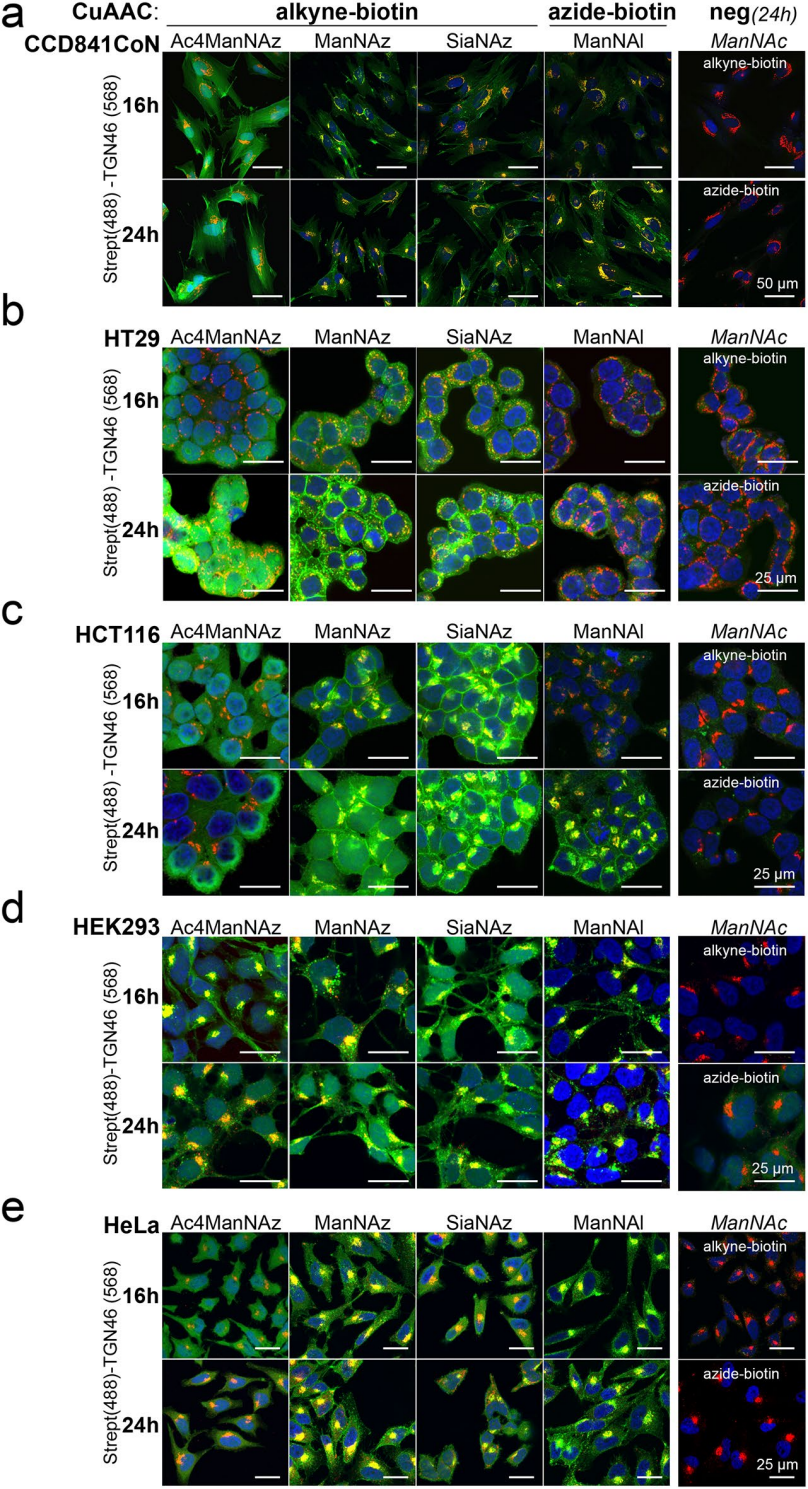
Confocal microscopy detection of labeled sialoglycoconjugates. Cells were incubated 16 h or 24 h with either the indicated analog or ManNAc (negative control). Then they were fixed and permeabilized before CuAAC with the appropriate biotin probe, followed by overnight incubation with anti-TGN46 (Golgi marker). Biotinylated proteins and TGN46 were revealed with Streptavidin-DyLight488 and AF568-conjugated secondary antibody, respectively. Nuclei were stained with DAPI before mounting. Two experiments were performed.

The labeling of sialylated GCs was monitored by confocal microscopy, 16 h or 24 h following metabolic incorporation of Ac_4_ManNAz, ManNAz, SiaNAz and ManNAl. SiaNAl was not included in this experiment because it gave only faint signal by flow cytometry. To compare the labeling of the different reporters within cells, we performed CuAAC with the azide-biotin or the alkyne-biotin probe on fixed and permeabilized cells. GCs were visualized using Streptavidin-DyLight488 (Strept). Sialylation occurs in the Golgi complex, making its organelle the first in which incorporated chemical reporters can be visualized onto GCs. Therefore, the Golgi marker TGN46 was used to colocalize the Strept signal in the Golgi (Fig. 3). We also quantified the intensity of fluorescence of sialylated GCs in and out of the Golgi to get an overview of the steady-state metabolic incorporation of each analog during the 16 or 24 h of continuous incubation (Fig. S5). A high integrated density (IntDen) of Strept "in the Golgi" (compared to the negative control) means that chemically modified CMP-NeuAc is efficiently transferred onto GCs by sialyltransferases. In contrast, a low and non-significant IntDen in the Golgi reflects a poor incorporation of the analog. The "out of Golgi" value reflects the steady-state trafficking of labeled GCs to the plasma membrane, their localization at the plasma membrane, but also their recycling in the endolysosomal system and the re-use of recycled non-natural CMP-NeuAc exported from the lysosomes. Because new chemical reporters are still being metabolized and processed in the Golgi apparatus in the meantime, the “in Golgi” value does not automatically decrease reciprocally.

As shown by confocal microscopy, metabolic incorporation of ManNAz, SiaNAz and ManNAl led to a strong fluorescent signal in the Golgi, as evidenced by the partial colocalization with TGN46 (Fig. 3) and the significant values of "in Golgi" IntDen of Streptavidin in the cells, except HT29 cells (Fig. S5). Also, fluorescent streptavidin was detected at the plasma membrane and in the cytoplasm (Fig. 3), leading to a significant increase of "out of Golgi" IntDen compared to the negative controls (Fig. S5). Green spots in cytoplasm might stand for vesicles from the secretory pathway, leading ultimately to the labeling of plasma membrane, and endocytic vesicles of recycling membrane glycoproteins. In HT29 cells, no specific signal was observed with ManNAl after 16 h of incorporation compared to the negative control (ManNAc, azide-biotin) (Fig. 3b). However, a slight colocalization of sialylated glycoconjugates with TGN46 was observed after 24 h, indicating that ManNAl was metabolized at a very low rate in these colon cancer cells (Fig. 3b).

Lastly, incubation of the colon cell lines with Ac_4_ManNAz led to a strong and diffuse nuclear and cytosolic labeling (Fig. 3a–c), as previously reported in another cancerous cell lines^19^. There was no colocalization of Strept signal with TGN46 in these cells, as confirmed by the quantification that showed that most of the signal was quantified as “out of Golgi” (Fig. S5a–c). This indicates that the signal obtained with Ac_4_ManNAz appears to be non-specific to the Golgi complex compared to the other analogs. Taken together, these data highlighted that ManNAz and SiaNAz reporters appear to be the best compromise for labeling of sialylated GCs into the Golgi and on cell surface of the human colon cells.

### Comparison of bioorthogonal labeling of sialylated glycoproteins by Western Blot

**Figure 4:**
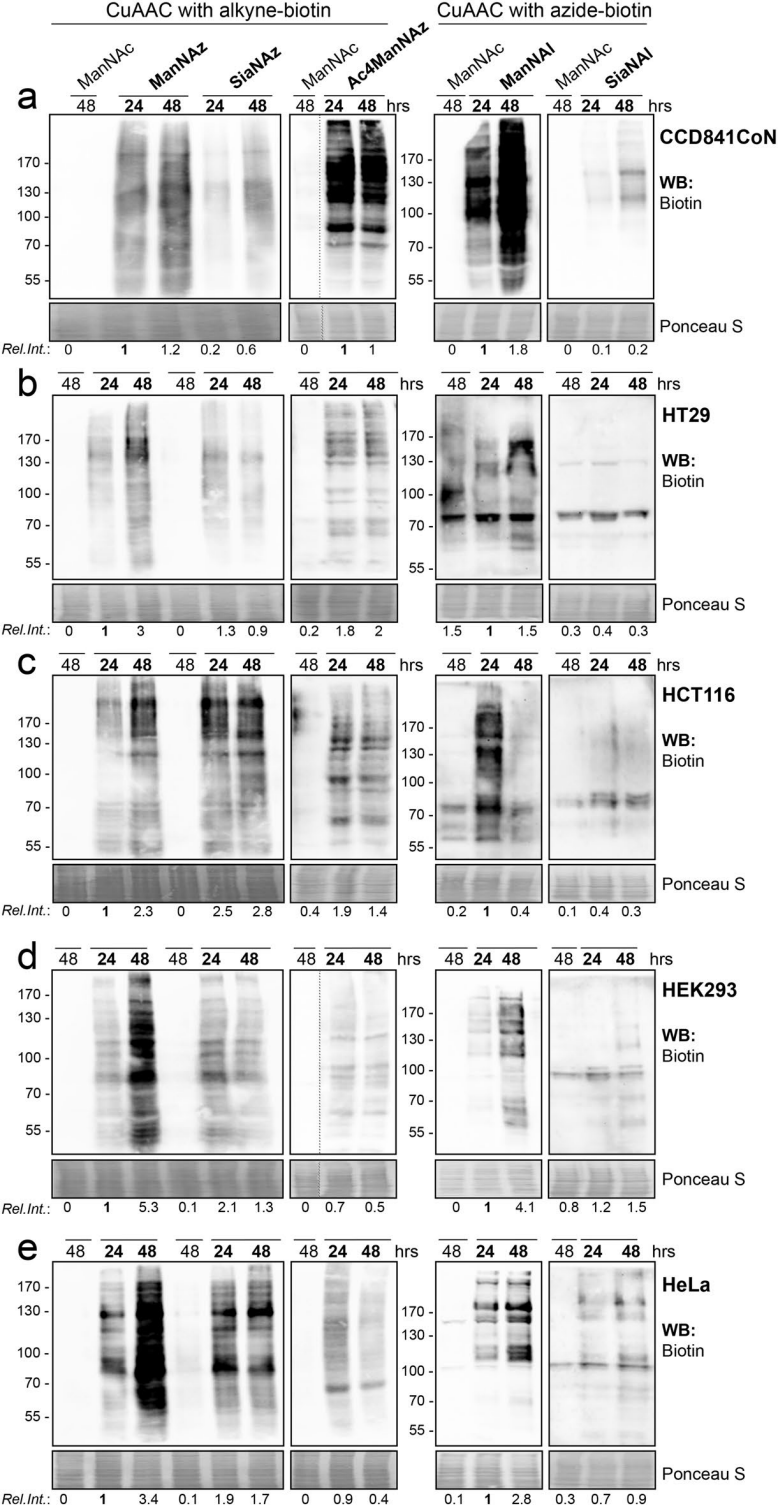
Comparison of the efficiency of metabolic incorporation of the analogs by Western blot. Unnatural monosaccharides were added in cell culture medium for the indicated times (at 500 µM, except for Ac_4_ManNAz, 50 µM). CuAAC was then performed onto whole cells using the appropriate biotin probe. Biotinylated glycoproteins were detected by Western blotting using anti-biotin-HRP. For each analog, similar time exposures are shown for all the cell lines, except for CCD841CoN cells for which shorter times have been chosen to avoid overexposure. Equal loading was confirmed by Ponceau red staining. The relative intensity (Rel. Int.) of each lane is indicated (biotin/Ponceau red), compared to the lane ‘24 h ManNAz’ or ‘24 h ManNAl’ for click chemistry with the alkyne probe or the azide probe, respectively. MW standards are indicated in kDa. Results are representative of three experiments.

We further investigated the discrepancies between metabolic incorporation of the unnatural alkyne- and azide-modified ManNAc/Neu5Ac by detecting the labeled glycoproteins (GPs) by Western blot. Cells were cultured for 24 or 48 h with the chemical reporters and click chemistry reaction was performed with the appropriate biotinylated probe onto whole cells. A large range of tagged sialoglycoproteins was detected in CCD841CoN cells whatever the analog, although the use of SiaNAz gave a fainter signal for the same time of exposure (Fig. 4a). Conversely, in HT29 cells, ManNAz was the most efficient chemical reporter for the labeling of sialylated GPs compared to the others: Faint signals were observed with SiaNAz and Ac_4_ManNAz, and no specific bands were detected with ManNAl and SiaNAl (Fig. 4b). In agreement with the flow cytometry analysis, incorporation of ManNAz, ManNAl, SiaNAz, and to a lesser extent Ac_4_ManNAz, gave a strong labeling of GPs in HCT116, HEK293 and HeLa cells, while only faint signals were obtained with SiaNAl for similar times of exposure (Fig. 4c–e).

Ac_4_ManNAz was used at 50 µM while the free hydroxyl analogs were used at 500 µM. To evaluate whether fainter signals obtained with Ac_4_ManNAz could be due to this large discrepancy, we evaluated the labeling of GPs in cells incubated for 48 h with 50 µM of all the analogs. Cells incubated with 50 µM ManNAc and 500 µM ManNAz stand respectively for the negative and positive controls (Fig. S6). We observed a stronger labeling of GPs with Ac_4_ManNAz compared with ManNAz and SiaNAz in HEK293 and HeLa cells, but not in the colon cell lines. No labeling was observed with 50 µM ManNAl and SiaNAl, whatever the cell lines (Fig. S6). These results indicate that high concentrations of free hydroxyl analogs are needed to force cell entry via active transport or endocytosis, whereas peracetylated reporter Ac_4_ManNAz penetrates the cells by passive diffusion. The strongest labeling of GPs was obtained with 500 µM ManNAz, therefore we used this reporter for the further experiments.

**Figure 5:**
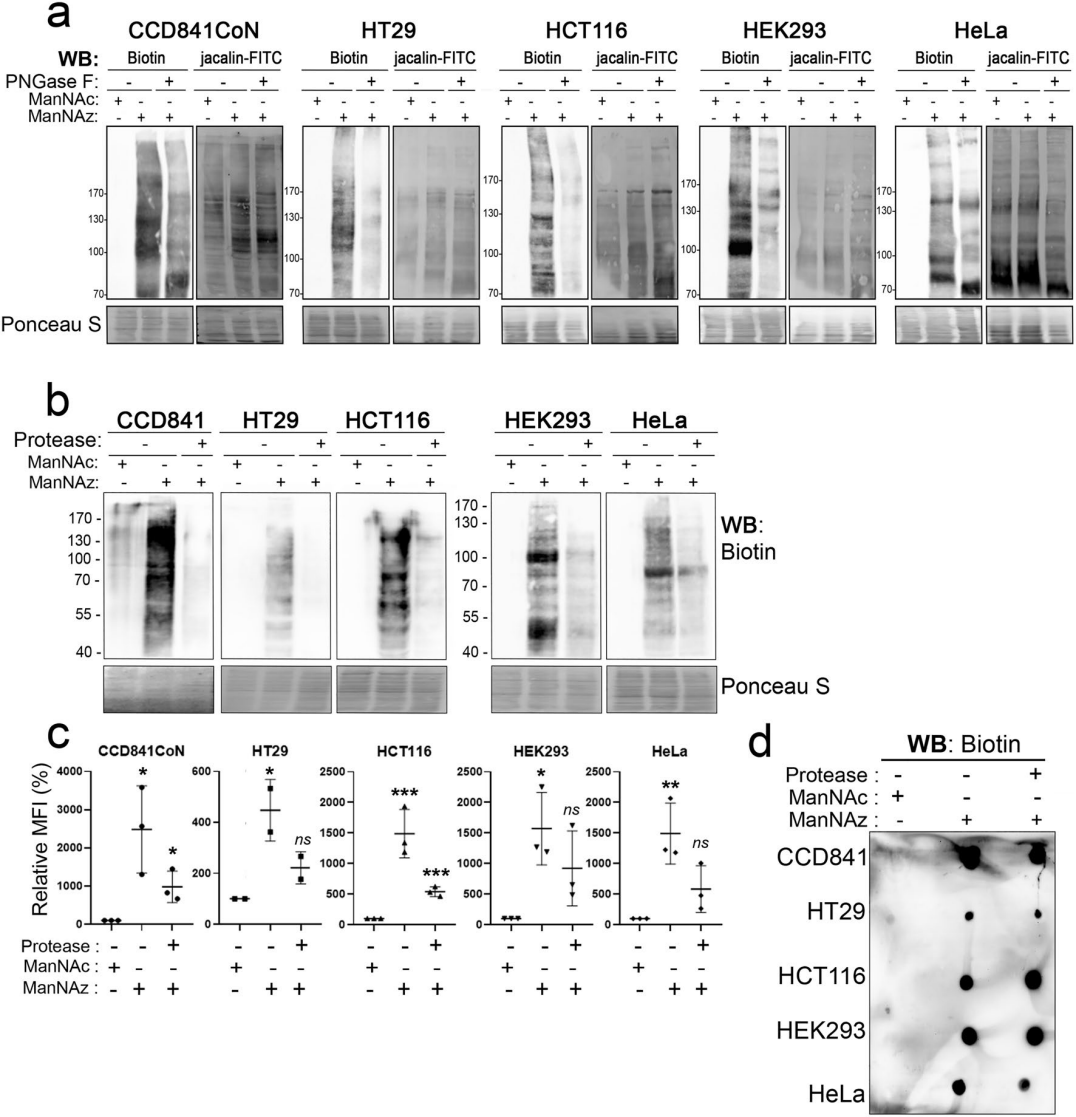
Sensitivity of labeling of glycoconjugates to PNGase F or protease treatment. Cells were incubated with 500 µM of either ManNAc (negative control) or ManNAz for 48 h before harvesting. **a**) After CuAAC with the alkyne-biotin, cells were lyzed and 30 µg of proteins were incubated with or without PNGase F before Western blotting using anti-biotin-HRP or Jacalin-FITC. **b**–**d**) Harvested cells were incubated for 15 min at 37 °C with or without the protease. Then CuAAC was performed onto cell pellets with either the alkyne-biotin probe (**b, d**) or the alkyne-AF488 probe for flow cytometry analysis (**c**). Labeled glycoproteins (**b**) or extracted gangliosides (**d**) were detected by Western blotting using anti-biotin-HRP. Results are representative of at least two experiments. *** *p* ≤ 0.005, ** *p* ≤ 0.01, * *p* ≤ 0.05; *ns*, non-significant (compared to the negative control).

We next examined the relative labeling of *N*- and *O*-glycoproteins using ManNAz in the various cell lines. CuAAC was performed as previously described with the alkyne-biotin probe before cell lysis. Lysates were then incubated with PNGase F to remove *N*-linked glycans from glycoproteins. The activity of PNGase F was first checked by lectin blotting using L-PHA which specifically binds β1,6-branched oligosaccharides of complex-type *N*-linked glycans. The L-PHA signal was rather totally lost in cell lysates treated with PNGase F compared with nontreated lysates, asserting the cleavage efficiency of PNGase F (Fig. S7). Then, treatment with PNGase F was applied on cell lysates following click chemistry reaction. The signal of biotin-tagged sialylated GPs detected by Western blot was reduced by approximatively 40% in CCD841CoN and by 70% in the other cell lines (Fig. 5a). Some of the remaining bands observed in the PNGase F-treated samples might match the signal of *O*-glycoproteins detected with jacalin, a lectin that recognizes the Galβ1,3GalNAc motif found on *O*-linked glycans (Fig. 5a).

Finally, to test whether ManNAz could be metabolized into both sialylated GPs and glycosphingolipids at the membrane of colon cells, harvested cells were incubated with bromelain, a widely acting protease. Then cells were washed and CuAAC was done using either the alkyne-biotin probe for Western blot (Fig. 5b, d), or the alkyne-AF488 probe for flow cytometry analysis (Fig. 5c). Loss of E-cadherin detection in bromelain-treated HT29 cells confirmed the cleavage activity of the protease towards cell surface GPs (Fig. S8a). Both α2,3 and α2,6 sialylation was affected by the protease, as evidenced by the decrease of cell surface fluorescence using respectively MAA and SNA lectins (Fig. S8b). We showed that detection of biotinylated sialoglycoproteins by Western blot was almost completely lost in cells incubated with bromelain compared to control cells (ManNAz, without protease) (Fig. 5b). However, flow cytometry analysis showed that the intensity of fluorescent tagged GCs was markedly reduced after treatment with bromelain compared to the controls: after bromelain treatment, cell surface fluorescence was reduced by approximately 50% (Fig. 5c). Sialylated glycosphingolipids (gangliosides) have been characterized in the three colon cell lines^25^, and in HEK293 and HeLa cells^32^. To assess whether the remaining cell surface fluorescence after protease treatment could be attributed to gangliosides, we performed lipid extraction after CuAAc, and glycolipids were spotted on PDVF membrane. Dot-blot using anti-biotin showed that labeled gangliosides were detected in samples from bromelain-treated or not treated cells (Fig. 5d). This indicates that both GPs and glycosphingolipids were labeled using ManNAz, as previously shown for example with Ac_4_ManNAz in Caco-2 colon cancer cells^33^, or SiaNAl in human fibroblasts^8^.

## Discussion

Previous studies reported that metabolic incorporation of alkyne- and azide- modified monosaccharides displays a large discrepancy, but most of these studies used peracetylated precursors. For example, peracetylated GlcNAz and GalNAz are more easily metabolized than their respective alkyne counterparts, and the efficiency of their metabolic incorporation is cell-type specific ^34^. In contrast, the metabolic conversion of Ac_4_ManNAl into SiaNAl is more efficient than Ac_4_ManNAz into SiaNAz in several cell lines^12^. Here, we used the non-protected analogs ManNAz, ManNAl, SiaNAz or SiaNAl and the peracetylated Ac_4_ManNAz to investigate the efficiency of labeling of cell surface sialoglyconjugates in three human colon cell lines in which we previously carried out glycomic analysis^25^, and compare them to the widely used HEK293 and HeLa cells. First, our results emphasize the cell line-dependent capability to metabolize the unnatural monosaccharides targeting sialic acid biosynthesis, as previously reported with Ac_4_ManNAz and other synthetic ManNAc analogs^12,19,33,35,36^. HEK293 and HeLa efficiently metabolize the five unnatural analogs. In contrast, CCD841CoN cells, HT29 and HCT116 colon cells display a marked difference in their ability to use the chemically modified ManNAc and Neu5Ac reporters, even though they derive from the same organ. Also, switching the azide and alkyne bioorthogonal tags highly impacts their metabolic incorporation. The non-immortalized and fetal colon CCD841CoN cells are the most promiscuous ones since they efficiently metabolize any of the analogs, whatever the tag used. Conversely, the colon adenocarcinoma cells HT29 poorly metabolize ManNAl and SiaNAl reporters in comparison with ManNAz and SiaNAz.

Impairment in the metabolic incorporation of the alkyne-modified precursors in HT29 cells might be due to a defect in the late steps of sialylation. It is unlikely to be due to a low activity of CMAS towards alkyne-substrates to produce CMP-Neu5Ac derivatives. Indeed, we previously showed that the activity of a recombinant bacterial CMAS was as effective for SiaNAz and SiaNAl as for Neu5Ac^37^. It is generally accepted that small modifications at C-5 and C-9 of the sialic acid moiety are well tolerated by sialyltransferases^7,12,38^. However, we cannot exclude that the activity rate of some sialyltransferases could be affected by the alkyne tag on the unnatural CMP-Neu5Ac. Indeed, it has been evidenced that the activity of ST8Sia-II, but not ST8Sia-IV, is affected by the use of C-5 modified sialic acids for the polysialylation of neural cell adhesion molecule (NCAM) in cells^39^. This is also the case for ST6Gal-I and ST3Gal-IV whose in vitro activity is differentially affected by C-9 modified neuraminic acids^40^.

Strikingly, HT29 is the only cell line used in this work to express two isoforms of GNE, GNE1 and GNE2. GNE2 has additional N-terminal 31 amino-acids compared to the ubiquitously expressed GNE1 (commonly referred to as GNE). GNE2 displays a drastically reduced epimerase activity and is found only as a dimer^30^. In contrast, GNE1 exists in different oligomeric states: the hexameric state is the fully functional active form of GNE in vitro, but how the oligomerization of GNE affects its functional activity into cells is not yet understood^29,30^. Interestingly, in vitro assays showed that GNE has a strongly reduced kinase activity towards ManNAl (ManNPoc) or ManNBut, compared to ManNAc^41^. In HT29 cells, the presence of GNE2 into the oligomeric complexes of GNE might change the promiscuous kinase activity of GNE towards ManNAl. Further investigations are needed to explore this hypothesis.

So far, Ac_4_ManNAz has been widely used for MOE targeting sialylation. Here we show that ManNAz is a good alternative for the labeling of cell surface sialylated GCs, especially for the colon cell lines whose proliferation is sensitive to high concentration of Ac_4_ManNAz (≥ 100 µM). It may come from the perturbation of intracellular pH due to acetic acid release into cells, and the cross-reactivity with cysteine residues of cytosolic proteins^23,28^. In addition, Ac_4_ManNAz metabolic incorporation results in a diffuse fluorescence in the cytoplasm and nucleus of HT29 and HCT116 cells. In contrast, distinct subcellular compartments such as Golgi apparatus are clearly labeled in colon cells treated with ManNAz. This may reflect the marked difference of metabolism kinetics of these two compounds. Indeed, ManNAz enters cells by an active transport mechanism that remains to be identified^9^ but is then directly incorporated into the sialic acid de novo metabolic pathway. On the contrary, Ac_4_ManNAz enters by passive diffusion, but requires non-specific cytosolic esterase activity to be fully deacetylated before being incorporated in the metabolic pathway. Diffuse cytoplasmic staining and relatively low labeling of GCs observed in colon cancer cells may come from an inefficient and/or partial deacetylation of Ac_4_ManNAz, which might perturb its metabolic incorporation and even induce cytotoxicity in these cells. As previously performed in cultured cells and organisms, longer incubation period could address this issue^12^.

Another hypothesis regarding the diffuse staining of labeled GCs into cells is supported by a recent study showing that incubation of human fibroblasts with Ac_4_ManNAl (Ac_4_ManNPoc) resulted in biosynthesis of not only CMP-SiaNAl, but also UDP-HexNAl^42^. The latter may come from the hijacking of unnatural ManNAc by GlcNAc-2-epimerase (RENBP), leading to the biosynthesis of chemically modified UDP-GlcNAc through the salvage pathway^43^ (Fig. 1). Cytosolic UDP-GlcNAz could be further used for *O*-GlcNAc modification proteins catalyzed by the nuclear and cytosolic enzyme, *O*-GlcNAc transferase (OGT)^23^. Interestingly, OGT is overexpressed in colon cancer cells^24^; this could explain in part the higher cytosolic and nuclear signal observed in the two cancer cell lines cultured with Ac_4_ManNAz, compared to the non-cancerous CCD841 cell line. It is worth noting that the low relative abundance of unnatural UDP-GlcNAc compared to its natural counterpart may not be sufficient to detect the incorporation of modified GlcNAc into the *N*- and *O*-linked glycans on cell surface, as evidenced by a glycomic study in prostate cancer cells cultured with Ac_4_ManNAz^44^.

Numerous unnatural ManNAc and Neu5Ac analogs are available to study sialylation in cells and organisms using MOE. However, as highlighted in this work, caution should be taken to carefully choose the chemical reporter for a given cellular model. Among the five reporters that we tested in this study, ManNAz is the most appropriate analog to label sialylated GCs that are being processed into cells and exposed at the cell surface of human colon cells. Also, the azido group offers the great advantage to be converted to an amine group in presence of a reducing agent^33,44^, thus facilitating the analysis of metabolically modified glycans by mass spectrometry.

## Methods

### Materials

Stock solutions (50 mM) of the chemically modified monosaccharides were prepared in PBS, except for Ac_4_ManNAz which was dissolved in DMSO. Acetylene-polyethylene glycol (PEG)4-Biotin (Alk-biotin, CLK-TA105, 10 mM in DMSO) and AF594-Picolyl-Azide (CLK-1296, 2 mM in DMSO) were purchased from Jena Bioscience (Jena, Germany). Azide-PEG3-biotin (Az-biotin, 762024, 10 mM in DMSO), Azide-fluor 545 (760757, 2 mM in DMSO), Fluor 488-Alkyne (761621, 2 mM in DMSO) were from Merck-Sigma-Aldrich. Streptavidin DyLight® 488 and lectins (MAA-II-FITC, FL-1344; SNA-FITC, FL-1301; Jacalin-FITC, FL-1151; L-PHA-biotin, B-1115) were from Vector Laboratories (Eurobio, Les Ulis, France).

### Chemical synthesis of unnatural monosaccharides

ManNAl (*N*-(4-pentynoyl mannosamine) and SiaNAl (*N*-(4-pentynoyl) neuraminic acid) were synthesized as previously described^9^.

### Synthesis of ManNAz (*N*-azidoacetylmannosamine)

2-azidoacetic acid (117 mg, 1.16 mmol, 1 eq) was dissolved along with DIC (*N*–*N’*-diisopropylcarbodiimide, 175 mg, 1.392 mmol, 1.2 eq), HOBt (hydroxybenzotriazole, 195 mg, 1.276 mmol, 1.1 eq) and DIPEA (*N*,*N*-diisopropylethylamine, 141 mg, 1.392 mmol, 1.2 eq) in DMF (dimethylformamide, 20 mL), D-mannosamine hydrochloride (250 mg, 1.16 mmol, 1 eq) was added and the reaction was stirred at room temperature (r.t.) for 19 h under argon. The total consumption of mannosamine was assessed by silica thin layer chromatography (TLC) (CH_2_Cl_2_/MeOH 9:1 v/v) before solvent removal under reduced pressure. The reaction crude was purified by silica flash column chromatography (50 µm, 40 g, dry load, CH_2_Cl_2_/MeOH 95:5 v/v), fractions containing the product were gathered and concentrated under reduced pressure, affording ManNAz as a white powder (175 mg, 0.67 mmol, 57%).

^1^H NMR (300 MHz, D_2_O) δ = 5.10 (d, *J* = 1.0 Hz, 1H, H1 α), 5.01 (d, *J* = 1.4 Hz, 1H, H1 β), 4.46 (dd, *J* = 4.0 Hz, 1.4 Hz, 1H, H2 β), 4.33 (dd, *J* = 4.4 Hz, 1.0 Hz, 1H, H2 α), 4.10–3.98 (m, 5H, H3 α + H8 αβ), 3.88–3.71 (m, 5H, H5 α + H3 β + H6 αβ), 3.56 (t, *J* = 9.5 Hz, 1H, H4 α), 3.46 (t, *J* = 9.8 Hz, 1H, H4 β), 3.38 (ddd, *J* = 9.8 Hz, 4.7 Hz, 2.1 Hz, 1H, H5 β).

^13^C NMR (75 MHz, D_2_O) δ = 171.87 (s, C7 α), 170.97 (s, C7 β), 92.89 (s, C1 α), 92.83 (s, C1 β), 76.40 (s, C5 β), 72.03 (s, C3 β), 71.97 (s, C5 α), 68.82 (s, C3 α), 66.76 (s, C4 α), 66.52 (s, C4 β), 60.41 (s, C6 β), 60.39 (s, C6 α), 54.28 (s, C2 β), 53.37 (s, C2 α), 51.72 (s, C8 β), 51.65 (s, C8 α).

### Synthesis of Ac_4_ManNAz (1,3,4,6-tetra-*O*-acetyl-*N*-azidoacetylmannosamine)

ManNAz (80 mg, 0.31 mmol, 1 eq) and DMAP (*N*,*N-*dimethylaminopyridine, catalytic amount) were dissolved in pyridine (5 mL). Acetic anhydride (189 mg, 1.85 mmol, 6 eq) was added dropwise and the reaction was stirred at r.t. for 16 h under argon. The reaction crude was concentrated under reduced pressure and purified by silica flash column chromatography (50 µm, 40 g, cyclohexane/ethyl acetate 2:1 v/v). Fractions containing the product were gathered and concentrated under reduced pressure, affording Ac_4_ManNAz as a white powder.

^1^H NMR (300 MHz, CDCl_3_) δ = 6.64 (d, *J* = 8.9 Hz, 1H, H1), 6.56 (d, *J* = 9.2 Hz, 1H, H1′), 6.05 (d, *J* = 1.9 Hz, 1H, NH′), 5.89 (d, *J* = 1.6 Hz, 1H, NH), 5.35 (dd, *J* = 10.1 Hz, 4.2 Hz, 1H, H3′), 5.22 (t, *J* = 10.1 Hz, 1H, H4′), 5.17 (t, *J* = 9.8 Hz, 1H, H4), 5.06 (dd, *J* = 9.8 Hz, 3.8 Hz, 1H, H3), 4.73 (ddd, *J* = 8.9 Hz, 3.8 Hz, 1.6 Hz, 1H, H2), 4.62 (ddd, *J* = 9.3, 4.2, 1.9, 1H, H2′), 4.30–4.00 (m, 5H, H5' + H6 + H6' + CH_2_-N_3_ + CH_2_-N_3_′), 3.82 (ddd, *J* = 9.8 Hz, 4.5 Hz, 2.5 Hz, 1H, H5), 2.24–1.93 (m, 12H, OAc + OAc′).

^13^C NMR (75 MHz, CDCl_3_) δ = 170.67–166.55 (m, CO OAc+C1+C1′), 91.31 (s, CN), 90.26 (s, CN′), 73.40 (s, C5), 71.45 (s, C3), 70.27 (s, C5′), 68.86 (s, C3′), 65.10 (s, C4′), 64.93 (s, C4), 61.75 (s, C6′), 61.67 (s, C6), 52.62 (s, CH_2_-N_3_), 52.45 (s, CH_2_-N_3_′), 49.72 (s, C2), 49.26 (s, C2'), 20.90–20.56 (m, 4 × CH3 OAc).

### Synthesis of SiaNAz (*N*-azidoacetylneuraminic acid)

ManNAz (60 mg, 0.228 mmol, 1 eq) was dissolved in 600 µl PBS (pH 7.6) with sodium pyruvate (92 mg, 0.456 mmol, 2 eq) in a 5 ml capped tube. 5 units of Neu5Ac aldolase (Sigma-Aldrich, EC 4.1.3.3) were added and the reaction was stirred at 37 °C for 16 h. The formation of the product was checked by TLC (1-propanol, NH_3_, H_2_O 6:1:2,5) with resorcinol as revelating agent. The reaction crude was purified by anion exchange chromatography (BioRad, AG1X8; H_2_O 5CV, NH_4_HCO_3_ 0.05 M 5CV, NH_4_HCO_3_ 0.2 M 5CV). Fractions containing the product were identified by TLC as previously then gathered and concentrated under reduced pressure. The white powder obtained was desalted using size exclusion chromatography (P2) with ultrapure water as eluent. Fractions containing the product were gathered, concentrated under reduced pressure then freeze-dried, affording the product as a white powder (20.6 mg, 0.059 mmol, 26%).

^1^H NMR (300 MHz, D_2_O) δ 4.09–3.88 (m, 5H, H5 + H4 + H3 + H2), δ 3.78 (dd, *J* = 11.2 Hz, 2.2 Hz, 1H, H8_cis_), δ 3.65 (ddd, *J* = 8.5 Hz, 4.1 Hz, 1.2 Hz, 1H, H7), δ 3.55 (dd, *J* = 11.2 Hz, 5.8 Hz, 1H, H8_trans_), δ 3.45 (dd, *J* = 9.2 Hz, 0.9 Hz, 1H, H6), δ 2.18 (dd, *J* = 12.5 Hz, 4.8 Hz,1H, H1_eq_), δ 1,75 (dd, *J* = 12.4 Hz, 11.8 Hz, 1H, H1_ax_).

^13^C NMR (75 MHz, D_2_O) δ 70.8 (CH, C5), δ 69.82 (CH, C7), δ 68.43 (CH, C6), δ 67.01 (CH, C2), δ 63.10(CH_2_, C8), δ 52.21(CH, C3), δ 51.9 (CH_2_, C4), δ 39.32 (CH_2_, C1).

### Cell culture

All the cell lines were purchased from ATCC. HT29 cells (derived from a colon adenocarcinoma), HCT116 cells (derived from a colon carcinoma), HeLa (derived from a cervix adenocarcinoma) and HEK293 human cells (derived from a human embryonic kidney) were maintained in Dulbecco’s modified Eagle’s medium (DMEM, 4.5 g/L glucose) (Bio West, Nuaille, France). CCD841CoN cells (primary cells from fetal normal colon) were maintained in Eagle’s Minimum Essential Medium (EMEM, 1 g/L glucose) (Lonza). All media were supplemented with 10% (v/v) fetal calf serum and 2 mM l-Glutamine (Lonza, Levallois-Perret, France). Cells were cultured at 37 °C in a 5% CO_2_-enriched humidified atmosphere.

### Metabolic incorporation of chemical reporters

CCD841CoN cells were seeded in 6-well plates (12 × 10^4^ cells/well). HT29 (10^5^/well), HCT116 (8 × 10^4^/well), HEK293 (5 × 10^4^/well) and HeLa (3.5 × 10^4^/well) were seeded in 12-well plates. One day later, ManNAc (500 µM, used as negative control) or the chemical reporter was added in cell culture medium (500 µM for all compounds except for Ac_4_ManNAz at 50 µM) for the indicated times. Then, cell monolayers were rinsed once with ice-cold PBS and cells were gently scrapped in 1 mL DPBS^−/−^ (DPBS without calcium and magnesium, Gibco™) before centrifugation in microtubes at 600 g and 400 g for 5 min at 4 °C for respectively CCD841CoN and cancer cells. Cells were then gently resuspended in 20 µL PBS.

### Click chemistry reaction

For Western blotting, CuAAC was performed onto cells using either 50 µM azido-PEG3-biotin or alkyne-PEG4-biotin diluted in a freshly prepared click buffer containing 100 mM K_2_HPO_4_, 150 µM CuSO_4_ (stock solution 15 mM), 300 µM BTTAA (stock solution 6 mM), 2.5 mM sodium ascorbate (stock solution 100 mM) (80 µl/tube). For the negative control, click reaction was performed on cells cultured with ManNAc. After the biorthogonal labelling one hour at r.t., 1 mL DPBS was added in the microtube, cells were centrifuged (1000 g for 5 min, at 4 °C) and then lysed in 35 µl of 2X Laemmli buffer. Samples were boiled before SDS-PAGE. For flow cytometry analysis, CuAAC was performed 45 min at r.t. in the dark using a fluorescent probe (10 µM AlexaFluor® 594-picolyl-azide or 15 µM alkyne-AlexaFluor® 488) in the click buffer. Cells were then washed three times in 1 mL DPBS and centrifuged (600 g for 5 min, at 4 °C). Finally, cells were resuspended in 100 µL DPBS and kept in ice until flow cytometry analysis.

### Protease treatment

To cleave plasma membrane proteins prior to the Cu(I)-catalyzed azide-alkyne cycloaddition (CuAAC), harvested and pelleted cells were treated with 0.1% (w/v) bromelain (B4882, Sigma-Aldrich) for 15 min at 37 °C in 100 µL or 150 µL of DPBS-0.1% (w/v) BSA, for respectively CCD841CoN and cancer cell lines^45^. As a negative control, cells were incubated without the protease in the same buffer. After incubation, cells were washed twice in 1 mL of DPBS and centrifuged (600 g*,* 5 min at 4 °C). Cell pellets were gently resuspended in 20 µL PBS before CuAAC.

### Extraction of glycolipids after protease treatment

Cells were cultured in T25 or T75 flasks with 500 µM ManNAc (negative control) or ManNAz for 48 h, and harvested as described above (approximatively 3 × 10^6^ CCD841CoN cells, 10^7^ HeLa cells, and 5 × 10^6^ cells for HT29, HCT116 and HEK293 cell lines).Cells were incubated for 15 min at 37 °C with or without bromelain (0.1% in 1 mL DPBS-0.1% BSA) before CuAAC with alkyne-PEG4-biotin (50 µM in 1 mL click reaction buffer per tube). Ten percent of the samples were set aside for SDS-PAGE and Western blotting. After the last centrifugation, cell pellets were frozen in 1 mL of water and lyophilized. Then glycolipids were extracted by the sequential addition of 1 mL CHCl_3_/CH_3_OH (2:1, v/v), CHCl_3_/CH_3_OH (1:1, v/v), and CHCl_3_/CH_3_OH/H_2_O (1:2:0.8), followed by centrifugation (2500 g, 20 min)^25^. Combined supernatants were dried under a nitrogen stream and the resulting glycolipids were dissolved in 100 µL CH_3_OH. PVDF membrane (Amersham™ Hybond™ P 0.45 PVDF, Dominique Dutscher, Illkirch, France) was dipped in methanol for 1–2 min and allowed to dry for a few minutes at r.t. To detect labeled sialylated glycolipids (gangliosides), samples (10 µL) were blotted onto the PVDF membrane and Western blotting was performed as described below.

### Cell surface labeling with lectin

After protease treatment, cells were incubated for 1 h at 4 °C with 100 µL of MAA-II-FITC or SNA-FITC, diluted at 20 µg/ml in PBS with 1% (w/v) BSA. Cells were washed three times in DPBS and resuspended in 100 µL of PBS before flow cytometry analysis.

### PNGase F treatment

*N*-glycans were released from cell surface glycoproteins using PNGase F (P0704, New England BioLabs, Evry, France), according to manufacturer’s instructions. Briefly, after CuAAC with the alkyne-PEG4-biotin, cell pellets were resuspended in water (10 µL per 10^4^ cells). 10× denaturing buffer was added to each sample and microtubes were incubated at 95 °C for 10 min. PNGase F digestion was performed on 10 µL of denaturated proteins (~ 30 µg) by adding 2 µL of 10× reaction buffer, 2 µL of 10% NP-40 solution, 1 µL of PNGase F and 4 µL of water (final volume 20 µl). After incubation at 37 °C for one hour, reaction was stopped by adding 5× Laemmli buffer and samples were heated at 95 °C for 7 min before SDS-PAGE.

### Western blotting

Proteins were separated by 8% SDS-PAGE and transferred onto nitrocellulose membrane (Amersham™Protran™ supported 0.45 µm NC, Dominique Dutscher, Illkirch, France). Membranes were stained with Ponceau Red (0.1% (w/v) Red Ponceau S in 5% (v/v) acetic acid) to confirm equal loading, washed with TBST (15 mM Tris pH 8, 140 mM NaCl, 0.05% Tween-20) before blocking in TBST with 5% (w/v) nonfat dry milk. Membranes were incubated overnight at 4 °C with peroxidase-conjugated monoclonal mouse anti-biotin (200–032-211, Jackson ImmunoResearch) (1:10,000) in blocking buffer. After four washes with TBST, chemiluminescence signal (SuperSignal West Pico Plus, Life Technologies, Courtaboeuf, France) was detected on a CCD camera (FUSION Solo, Vilber Lourmat, France) using the FUSION-Capt Solo software for acquisition and processing merge images. Western blot with the following antibodies were done as previously described^46^: GNE (ab189927, Abcam; 1:1,000), E-cadherin (sc-7870, Santa Cruz;1:1000), GAPDH (sc-32233, Santa Cruz; 1:2000), alpha-tubulin (sc-23948, Santa Cruz; 1:2000). For detection of *O*-linked glycoproteins, membranes were incubated overnight at 4 °C in blocking buffer (1% (w/v) BSA in PBS-Tween 0.05% (v/v)) before incubation with Jacalin-FITC (1:100) in blocking buffer for one hour at r.t. After washes, fluorescence was read with ImageQuant™ LAS 4000 (GE Healthcare). *N*-linked glycoproteins were revealed using L-PHA-biotin (1:500 in PBST with 1% BSA), followed by incubation with the anti-biotin-HRP. Densitometry analysis was done using the ImageJ® software.

### Flow cytometry analysis

Cell surface fluorescence was measured on a BD ACCURI C6 Plus flow cytometer (BD Biosciences, Rungis, France), either in the FL1 channel for labeling with the Alkyne-AF488 probe and lectin-FITC, or in the FL2 channel for labeling with the picolyl-azide-AF594 probe. Data were analyzed with FlowJo software (vX 0.7). Cell population was gated based on SSC/FSC dot plot to remove cell debris and doublets. The means of fluorescence intensity (MFI) were obtained for each condition (FL1A or FL2A), unspecific MFI was quantified from cells incubated with ManNAc (negative control). Raw values of MFI are indicated on the respective histograms in order to be able to compare the MFI obtained with a given fluorescent probe for the different analogs performed in the same experiment. Histograms with the relative MFIs compared to the negative controls are presented in Fig. S4 (mean ± standard deviation, n = 3).

### Immunofluorescence and confocal microscopy

Cells were seeded in 24-wells plate on a 10 mm glass coverslip (CCD841CoN, 25 × 10^3^/well; HT29, 30 × 10^3^/well; HCT116 and HEK293, 23 × 10^3^/well; HeLa 20 × 10^3^/well). One day later, chemical reporters were added in the cell culture medium for the indicated times. Then cells were gently washed in DPBS and fixed in 4% (w/v) paraformaldehyde (PAF) for 30 min at r.t. Coverslips were then washed three times in PBS (5 min) before quenching with 100 mM glycine for 20 min at r.t. After three washes with PBS, cells were permeabilized with 0.5% (v/v) Triton X100 in PBS for 10 min. After three washes in PBS, coverslips were incubated for 30 min at r.t. in the dark and under gentle agitation with 100 µl of the freshly prepared click reaction mix containing the appropriate biotinylated probe (50 µM). After three washes with PBS, immunofluorescence was performed as previously described with the rabbit polyclonal anti-TGN46 (1:100, AHP1586, BioRad, Marnes-la Coquette, France) and the goat anti-Rabbit IgG Alexa Fluor 568® (1:600)^46^. Streptavidin-DyLight®488 (1:600) was added at the same time to reveal click chemistry reaction. Nuclei were stained with DAPI (50 µg/mL) and coverslips were mounted on microscope slides using a drop of mounting medium (Dako mounting medium, Agilent). Images were acquired on a Nikon A1R confocal microscope (Apo 60X, NA1.4, oil immersion) using NIS-Elements software, and further analyzed with ImageJ® software.

We developed a home-made macro on Image J® to quantify the relative fluorescence signal of streptavidin in whole cell vs. Golgi apparatus (10.5281/zenodo.6778042). Briefly, a threshold value was first applied to confocal images based on the streptavidin channel intensity of each condition’s control to get rid of the background signal emanating from the binding of the streptavidin into cells (negative controls, i.e. CuAAC performed on cells cultured with ManNAc for 24 h). Regions of interest (ROI) of the whole cells were generated on the thresholded images. Golgi ROIs were created using the TGN46 detection channel. These ROIs allowed us to discriminate regions *in* and *out of* the Golgi apparatus, and to measure and export the streptavidin signal values (min, max and mean grey values, integrated densities) of these regions. Results are presented according to the mean of the integrated density (IntDen), representing the concentration of the signal of streptavidin in the ROI. Standard deviation was obtained from at least two images of two independent experiments.

### Statistical analysis

Unpaired/two-tailed *t*-test was used. *p*-values were calculated and reported accordingly (*** *p* ≤ 0.005, ** *p* ≤ 0.01, * *p* ≤ 0.05; ns, non-significant).

## Supplementary Information

Supplementary Legends.

Supplementary Figure 1.

Supplementary Figure 2.

Supplementary Figure 3.

Supplementary Figure 4.

Supplementary Figure 5.

Supplementary Figure 6.

Supplementary Figure 7.

Supplementary Figure 8.

Supplementary Information 10.

## Data Availability

The datasets generated for the image analysis are available from the corresponding author on request.
