# Supplementary material for: Switching azide and alkyne tags on bioorthogonal reporters in metabolic labeling of sialylated glycoconjugates: a comparative study
Source: Sci Rep. 2022 Dec 22;12:22129. doi: 10.1038/s41598-022-26521-3 (PMC9780200; doi:10.1038/s41598-022-26521-3)
Supplement: Supplementary file 1 — Supplementary Legends. [file 41598_2022_26521_MOESM1_ESM.docx]

**Switching Azide and Alkyne Tags on Bioorthogonal Reporters in Metabolic Labeling**

**of Sialylated Glycoconjugates: A Comparative Study.**

Scache Jodie^#^, Rigolot Vincent^#^, Lion Cédric, Mortuaire Marlène, Lefebvre Tony, Biot Christophe, Vercoutter-Edouart Anne-Sophie*

**Supplementary Information**

**Legend of supplementary figures**

**Supplementary Figure 1. Chemical structures of the non-natural ManNAc and Neu5Ac analogs used in this study.**

**Supplementary Figure 2**. **Dose-effect of Ac_4_ManNAz on proliferation of colon cell lines.** Cells were seeded in 12-well plate (75 x 10^3^ cells/well for CCD841CoN) or 24-well plate (50 x 10^3^ cells/well for HT29, 40 x 10^3^ cells/well for HCT116) for 24 hours before adding Ac_4_ManNAz at the indicated concentrations (µM). Viable cells were counted 48 hours later (Trypan Blue exclusion assay). *** p≤0.005, ** p≤0.01, * p≤0.05.

**Supplementary Figure 3. Expression of GNE by Western-blot.** Cells were incubated for 48 hours with ManNAc or ManNAz (500 µM) before cellular lysis and SDS-PAGE. GNE and α-tubulin (control loading) were detected by Western-blot. MW standards are indicated in kDa. Results are representative of two experiments.

**Supplementary Figure 4. Flow cytometry analysis of labeled sialoglyconjugates at cell surface**. Cells were incubated 24 hours or 48 hours with the analogs before CuAAC using the appropriate fluorescent probe. For the negative controls, CuAAC was performed on cells cultured with ManNAc. Histograms showing the relative mean of fluorescent intensity (MFI) for each of the cell lines and analogs ("100%" refers to the negative control). Mean and S.D. were obtained from three independent experiments. *** *p*≤0.005, ** *p*≤0.01, * *p*≤0.05 (compared to the negative control, unless indicated).

**Supplementary Figure 5. Quantification of labeling of glyconjugates into cells.** Labeled sialoglycoconjugates were detected by confocal microscopy after CuAAC and incubation with Streptavidin-DyLight488. Golgi was detected using an anti-TGN46 antibody. Quantification of the relative fluorescence signal quantification *in* (left panel) and *out* (right panel) of the Golgi was performed as described in Methods, using Image J software. The integrated density (IntDen) of streptavidin was reported for each chemical reporter. *** p≤0.005, ** p≤0.01, * p≤0.05.

**Supplementary Figure 6. Comparison of efficiency of metabolic incorporation of the analogs by Western blot.** Cells were incubated for 48 hours with 50 µM of either ManNAc (negative control) or chemically modified reporters (Ac_4_ManNAz, ManNAz, SiaNAz, ManNAl, SiaNAl). Incubation of cells with 500 µM ManNAz was used as positive control. Then CuAAC was performed with alkyne- or azide-biotin probe before cell lysis and SDS-PAGE. Labeled glycoproteins were revealed by chemiluminescence using an anti-biotin-HRP. Equal loading was confirmed by the Ponceau S staining of nitrocellulose membrane. MW standards are indicated in kDa. Results are representative of two experiments.

**Supplementary Figure 7. Detection of complex-type *N*-linked glycoproteins by L-PHA lectin blot after PNGase F treatment.** Cells were harvested and lysed. 30 µg of proteins were incubated with or without PNGase F for 1 hour at 37°C before SDS-PAGE. *N*-glycoproteins were revealed by Western blotting using the biotin-conjugated L-PHA lectin (β1,6 *N*-linked glycans), followed by incubation with anti-biotin-HRP antibodies. MW standards are indicated in kDa. Results are representative of two experiments.

**Supplementary Figure 8. Cell surface α2,3 and α2,6 sialylation was affected by protease treatment. a)** HT29 cells were incubated with or without ManNAz for 48 hours before harvesting. Cells were then incubated with or without the protease for 15 min at 37°C, before lysis in Laemmli buffer and SDS-PAGE. E-cadherin was detected by Western-blot, tubulin was used as control loading. MW standards are indicated in kDa. **b**) Cells were treated or not with the protease and cell surface sialylation was detected by flow cytometry using MAA-FITC and SNA-FITC lectins. Results are representative of two experiments
