# Supplementary figures and images for: Switching azide and alkyne tags on bioorthogonal reporters in metabolic labeling of sialylated glycoconjugates: a comparative study
Source: Sci Rep. 2022 Dec 22;12:22129. doi: 10.1038/s41598-022-26521-3 (PMC9780200; doi:10.1038/s41598-022-26521-3)

## Supplementary Figure 1

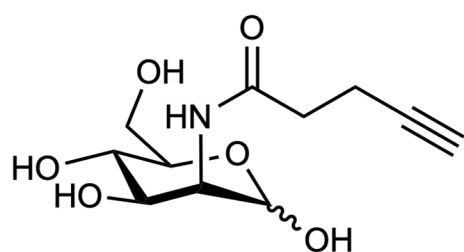

ManNAI

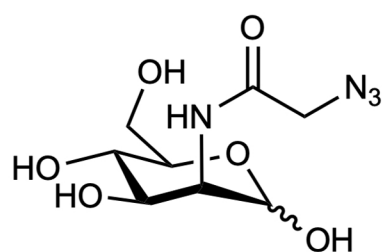

ManNAz

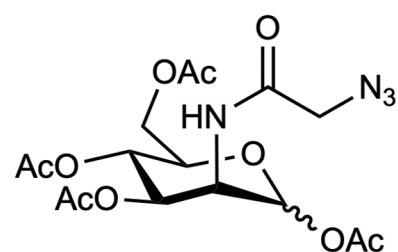

Ac<sub>4</sub>ManNAz

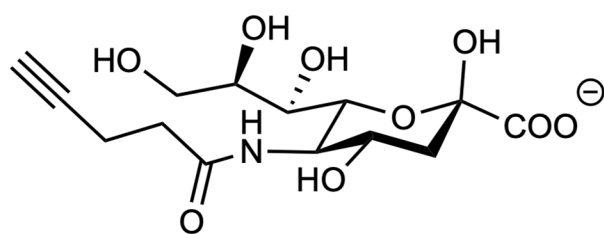

SiaNAI

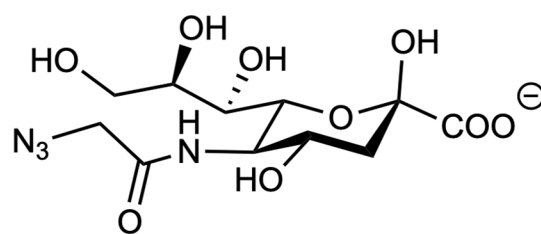

SiaNAz

Supplement: Supplementary file 2 — Supplementary Figure 1. [file 41598_2022_26521_MOESM2_ESM.pdf]

Supplementary Figure 2

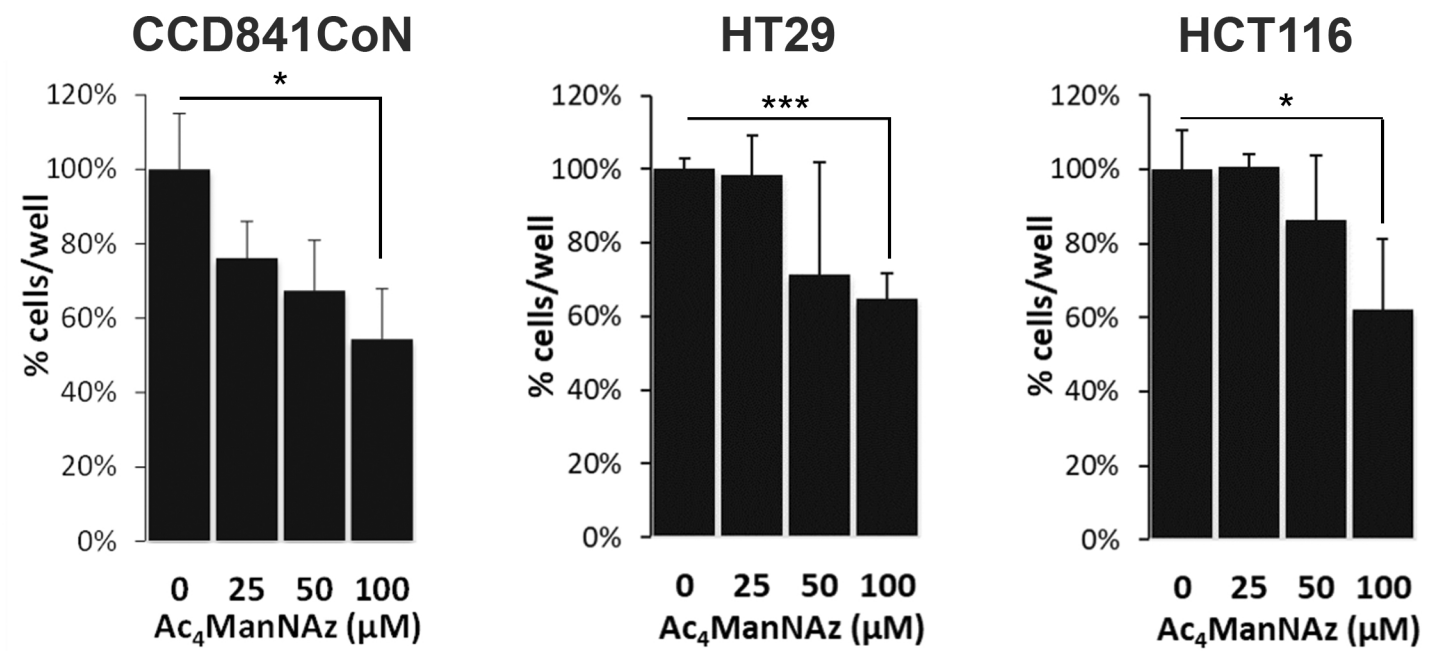

Supplement: Supplementary file 3 — Supplementary Figure 2. [file 41598_2022_26521_MOESM3_ESM.pdf]

Supplementary Figure 3

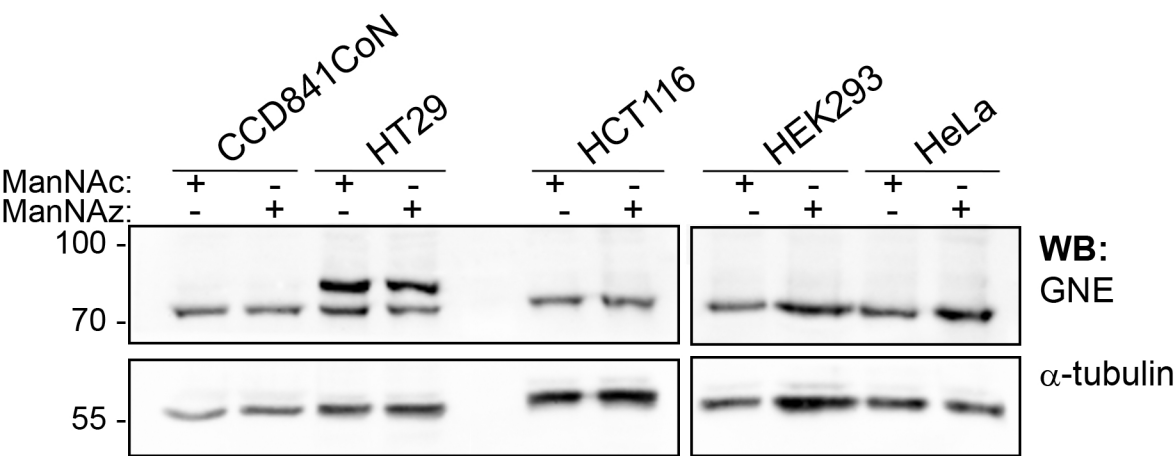

Supplement: Supplementary file 4 — Supplementary Figure 3. [file 41598_2022_26521_MOESM4_ESM.pdf]

**Supplementary Figure 4**

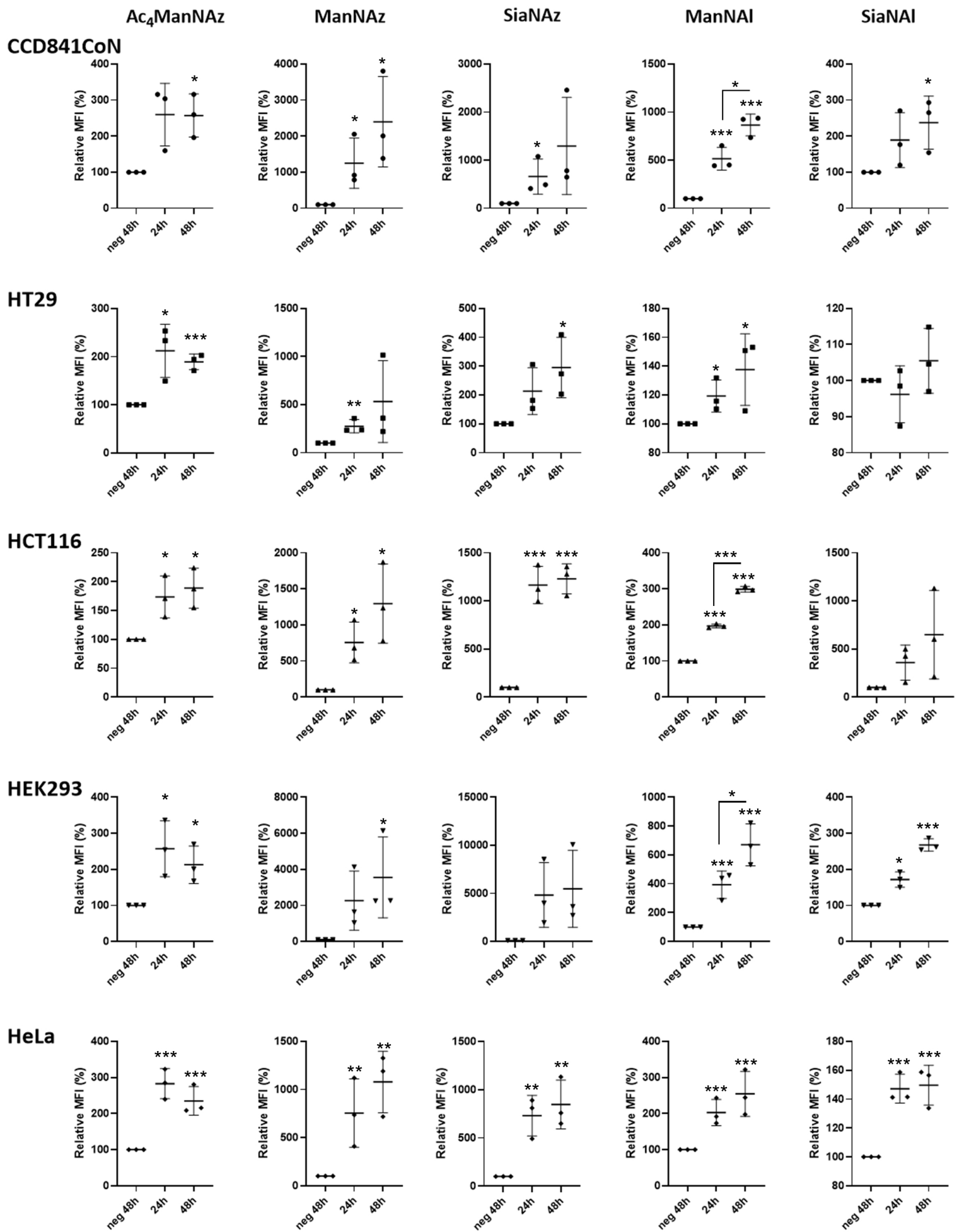

Supplement: Supplementary file 5 — Supplementary Figure 4. [file 41598_2022_26521_MOESM5_ESM.pdf]

Supplementary Figure 5

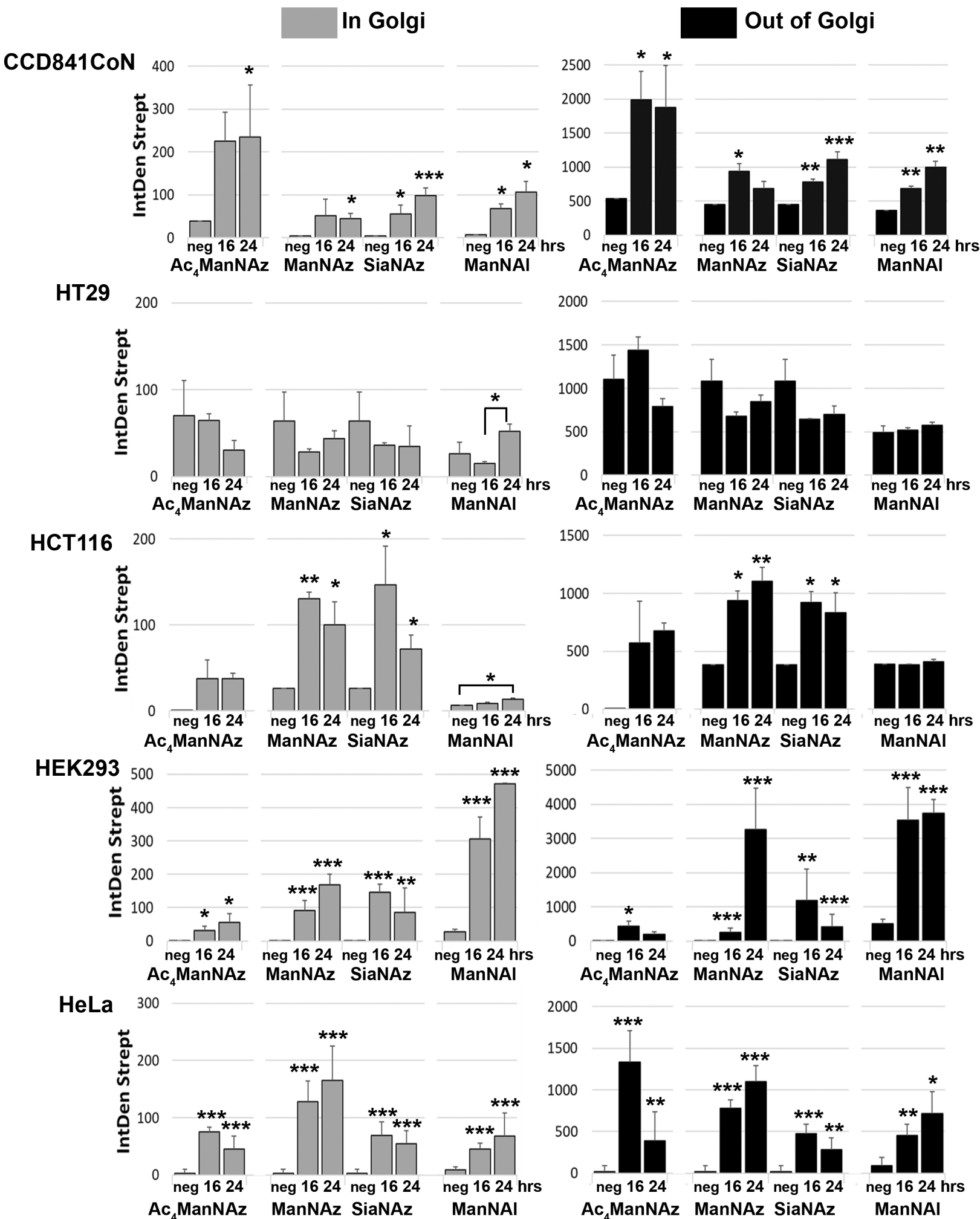

Supplement: Supplementary file 6 — Supplementary Figure 5. [file 41598_2022_26521_MOESM6_ESM.pdf]

## Supplementary Figure 6

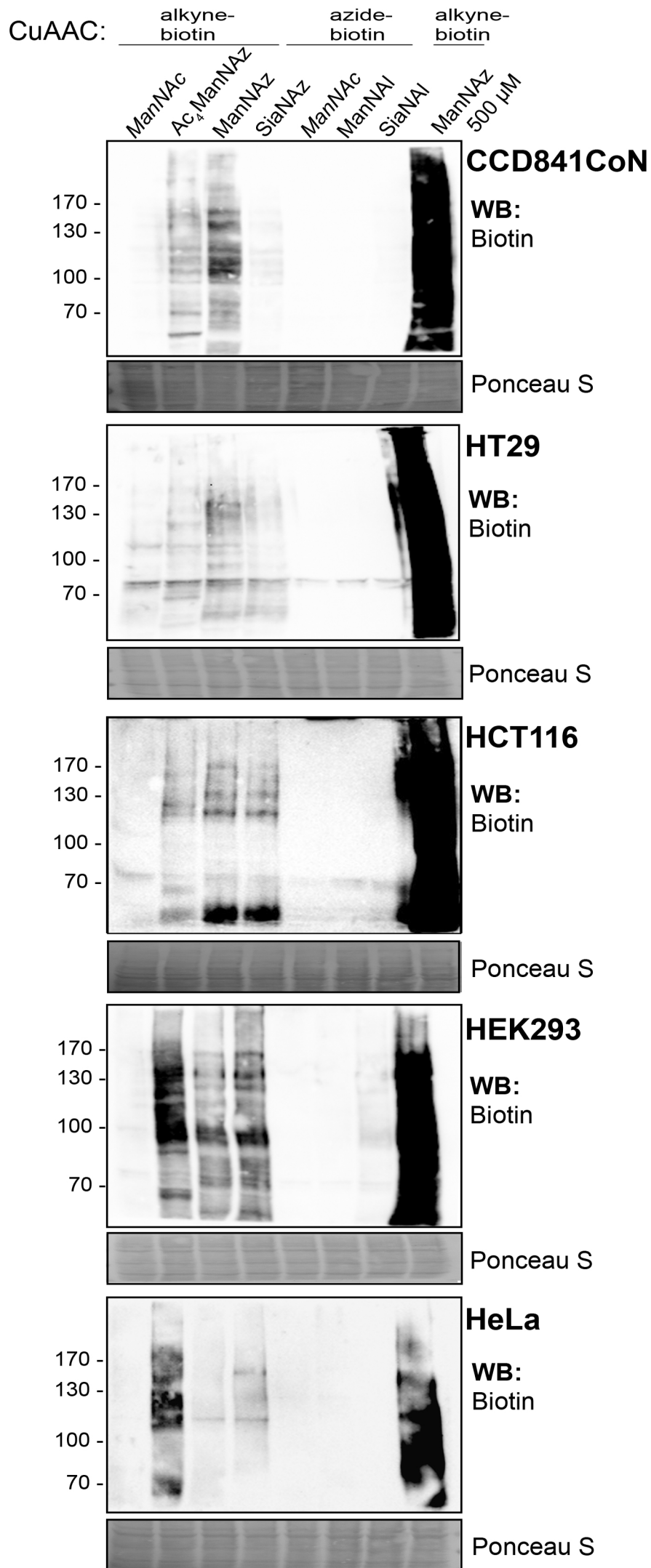

Supplement: Supplementary file 7 — Supplementary Figure 6. [file 41598_2022_26521_MOESM7_ESM.pdf]

Supplementary Figure 7

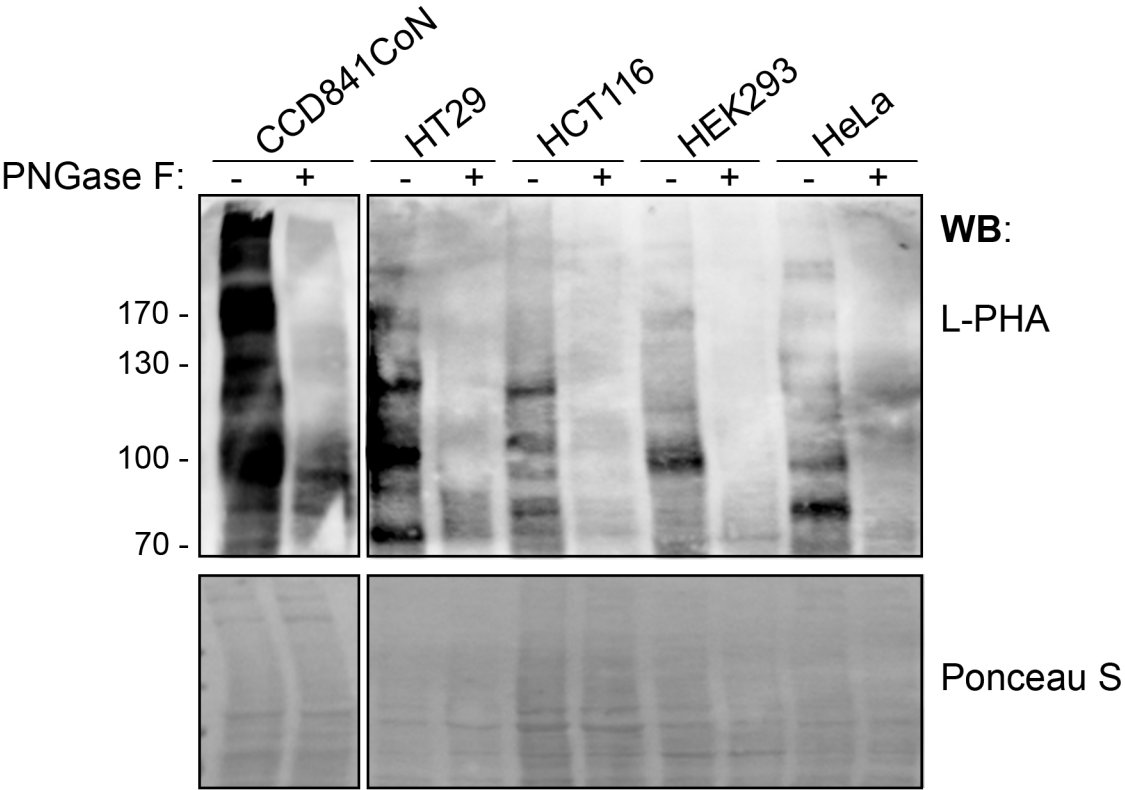

Supplement: Supplementary file 8 — Supplementary Figure 7. [file 41598_2022_26521_MOESM8_ESM.pdf]

Supplementary Figure 8

a

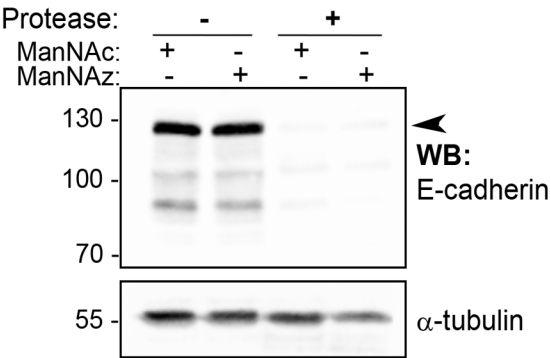

b

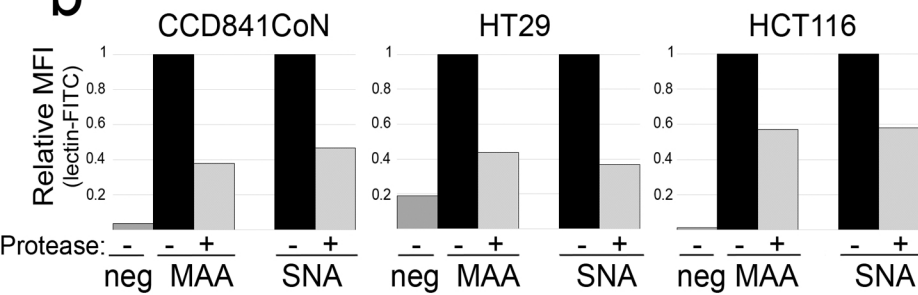

Supplement: Supplementary file 9 — Supplementary Figure 8. [file 41598_2022_26521_MOESM9_ESM.pdf]
