# Supplementary material for: Switching azide and alkyne tags on bioorthogonal reporters in metabolic labeling of sialylated glycoconjugates: a comparative study
Source: Sci Rep. 2022 Dec 22;12:22129. doi: 10.1038/s41598-022-26521-3 (PMC9780200; doi:10.1038/s41598-022-26521-3)

Supplementary Info: Figure 4, uncropped images (1/3)

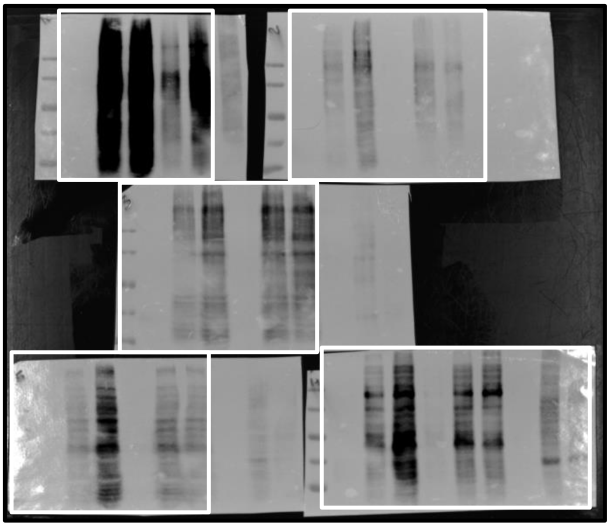

WB:
Biotin

Merge image with MW

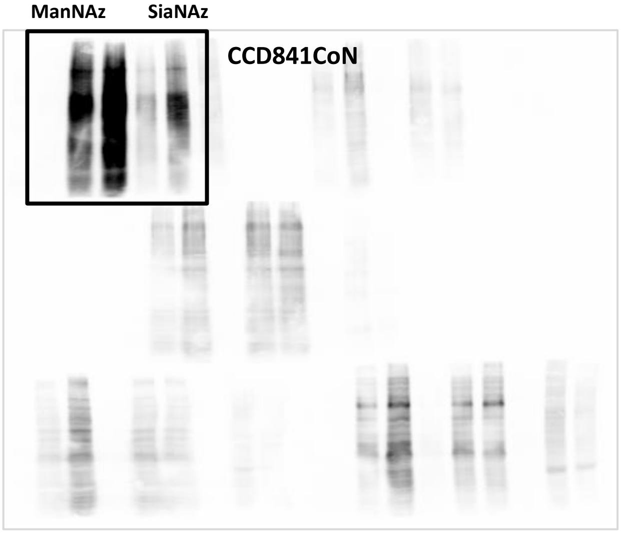

Exposure: 1 sec

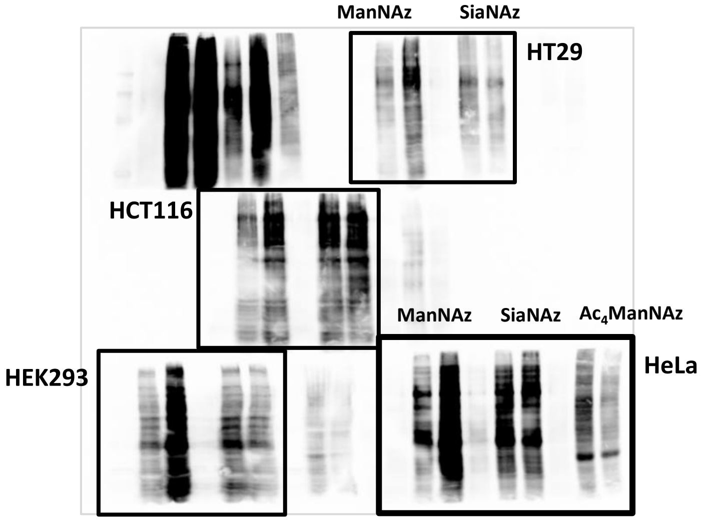

Exposure: 2 sec

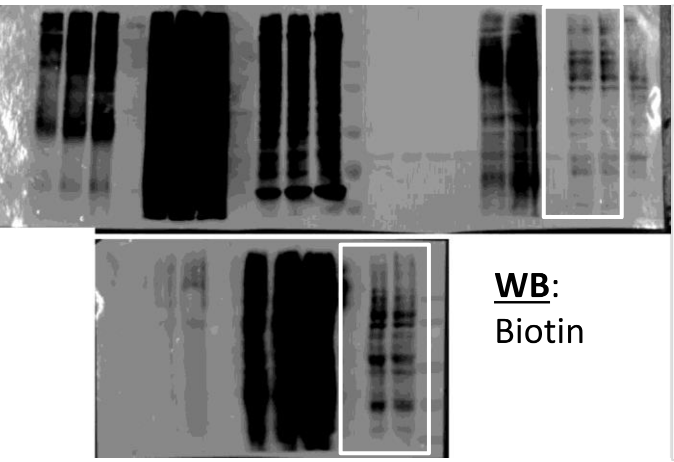

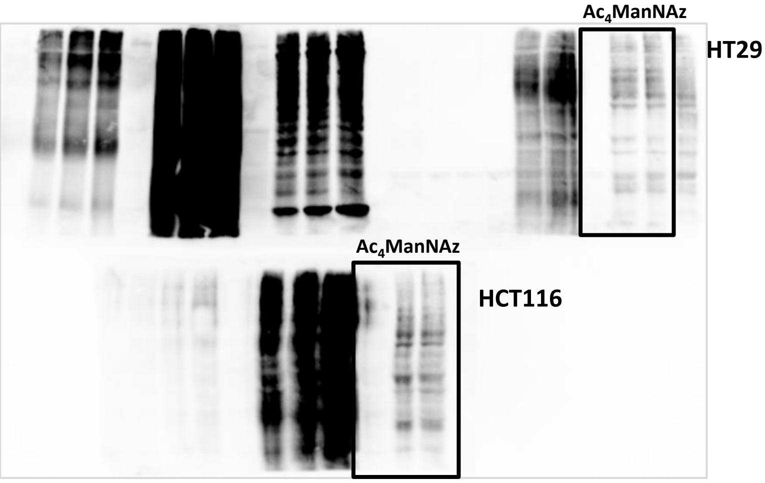

Exposure: 20 sec

Supplementary Info: Figure 4, uncropped images (2/3)

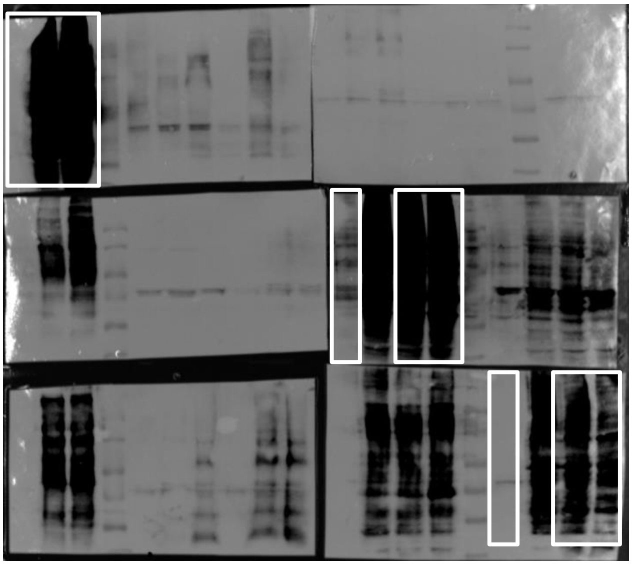

Merge with MW (30 sec)

WB: Biotin

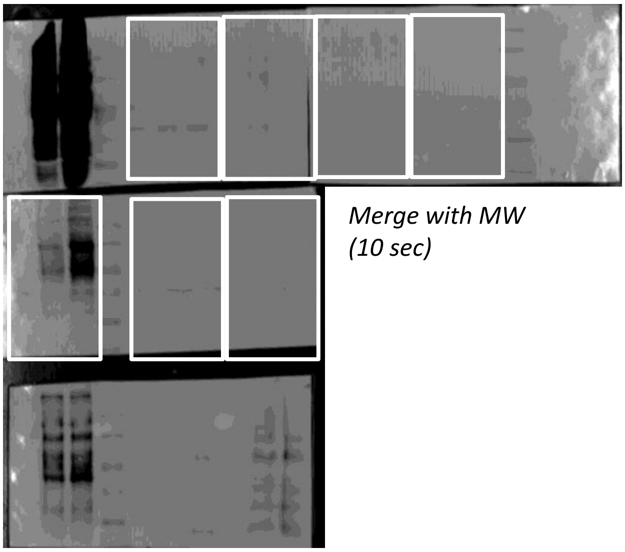

Merge with MW (10 sec)

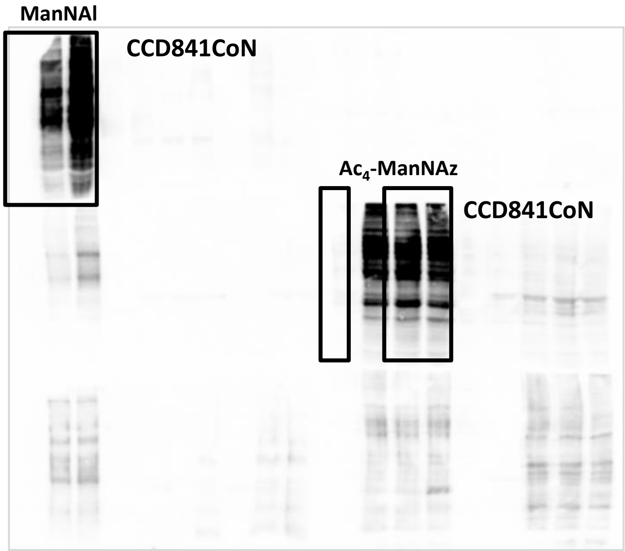

Exposure: 10 sec

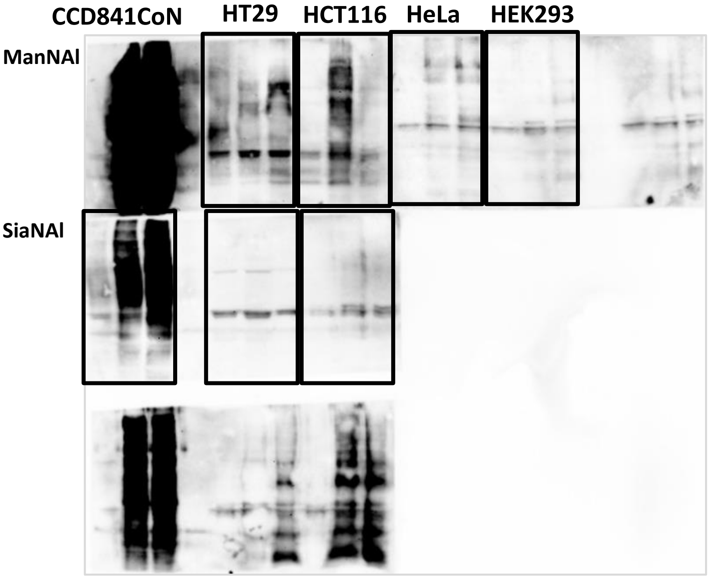

Exposure: 5 min

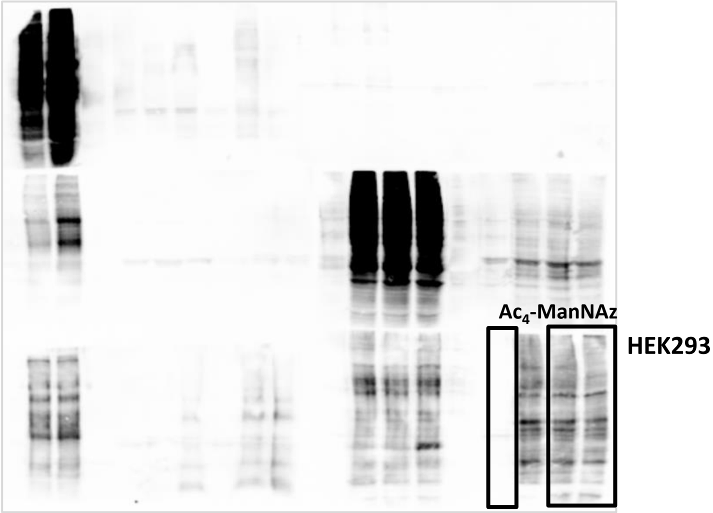

Exposure: 30 sec

Supplementary Info: Figure 4, uncropped images (3/3)

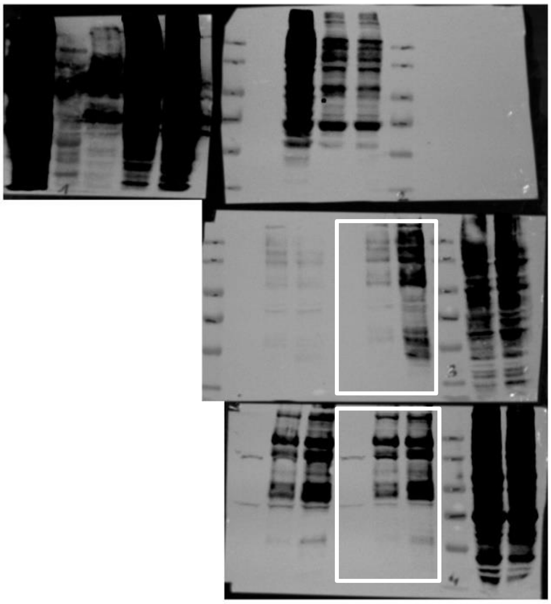

Merge with MW (20 sec)

WB: Biotin

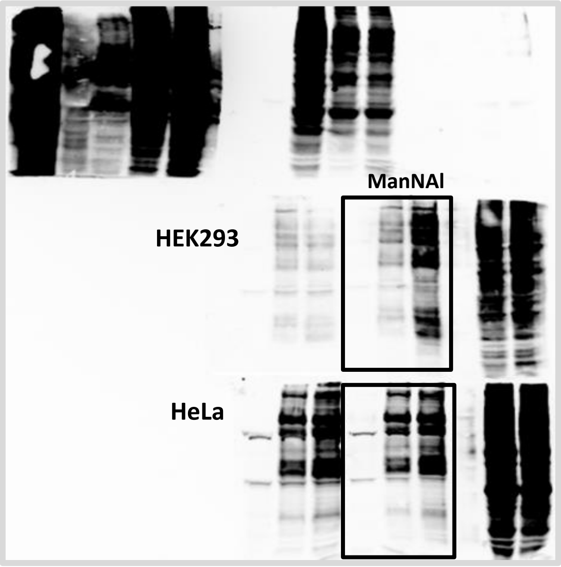

Exposure: 20s

Supplementary Info: Figure 5a,b, uncropped images

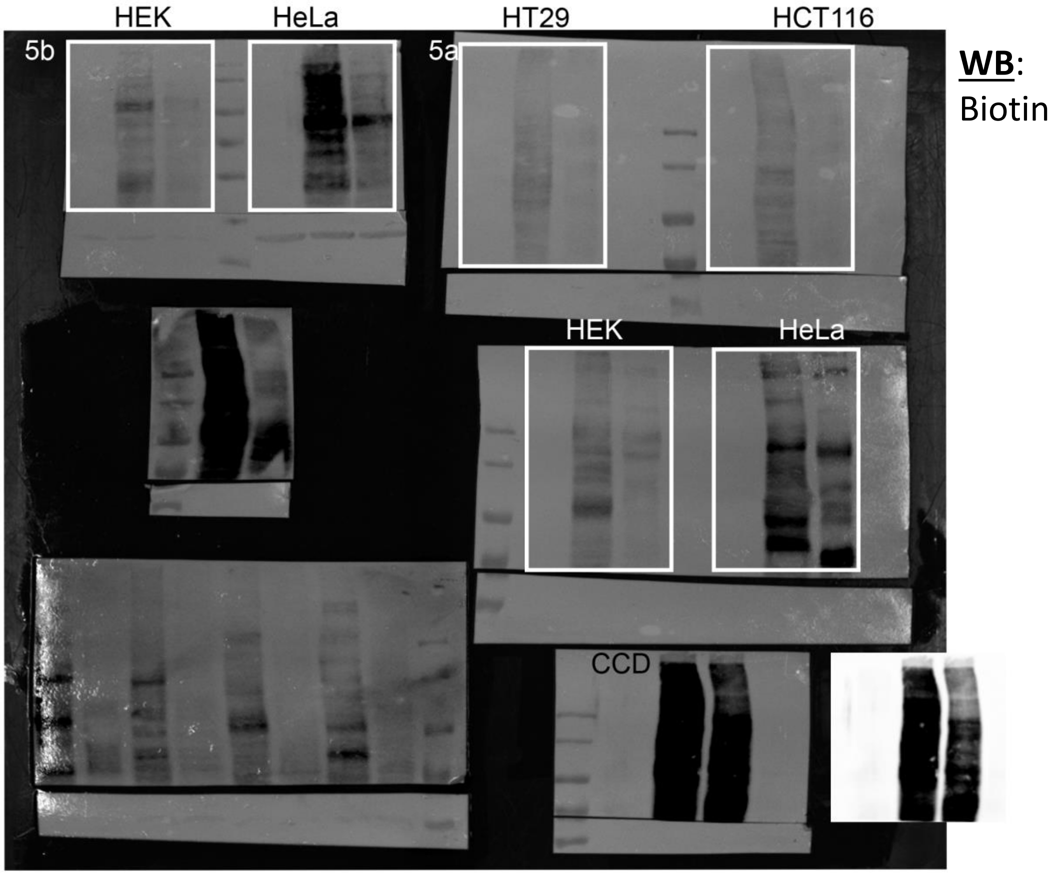

WB: Biotin

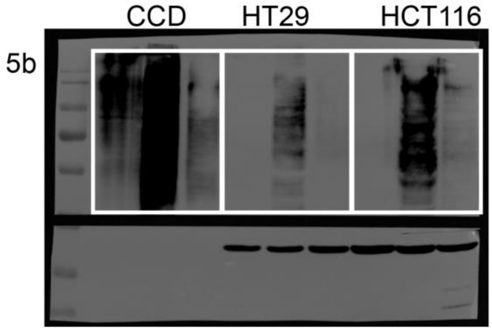

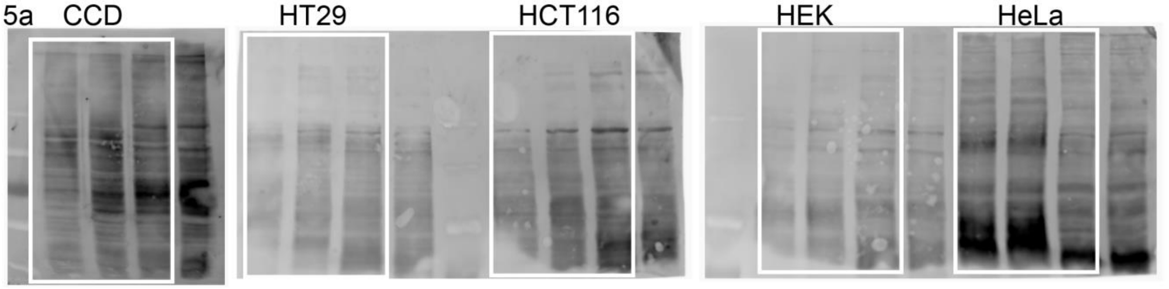

WB: Jacalin-FITC

Supplementary Info: Figure 5d, uncropped images

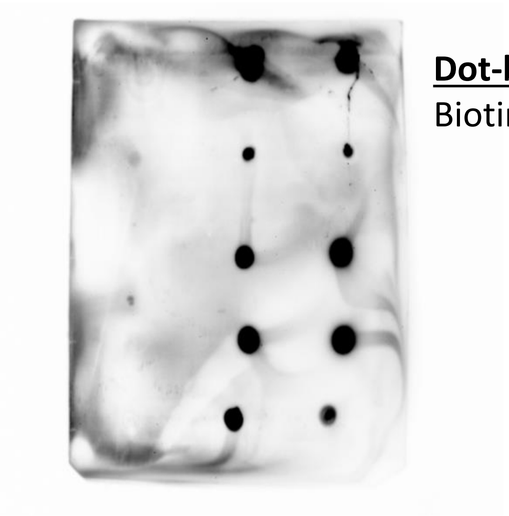

<u>Dot-blot</u>:
Biotin

Supplementary Info: Figure S3, uncropped images

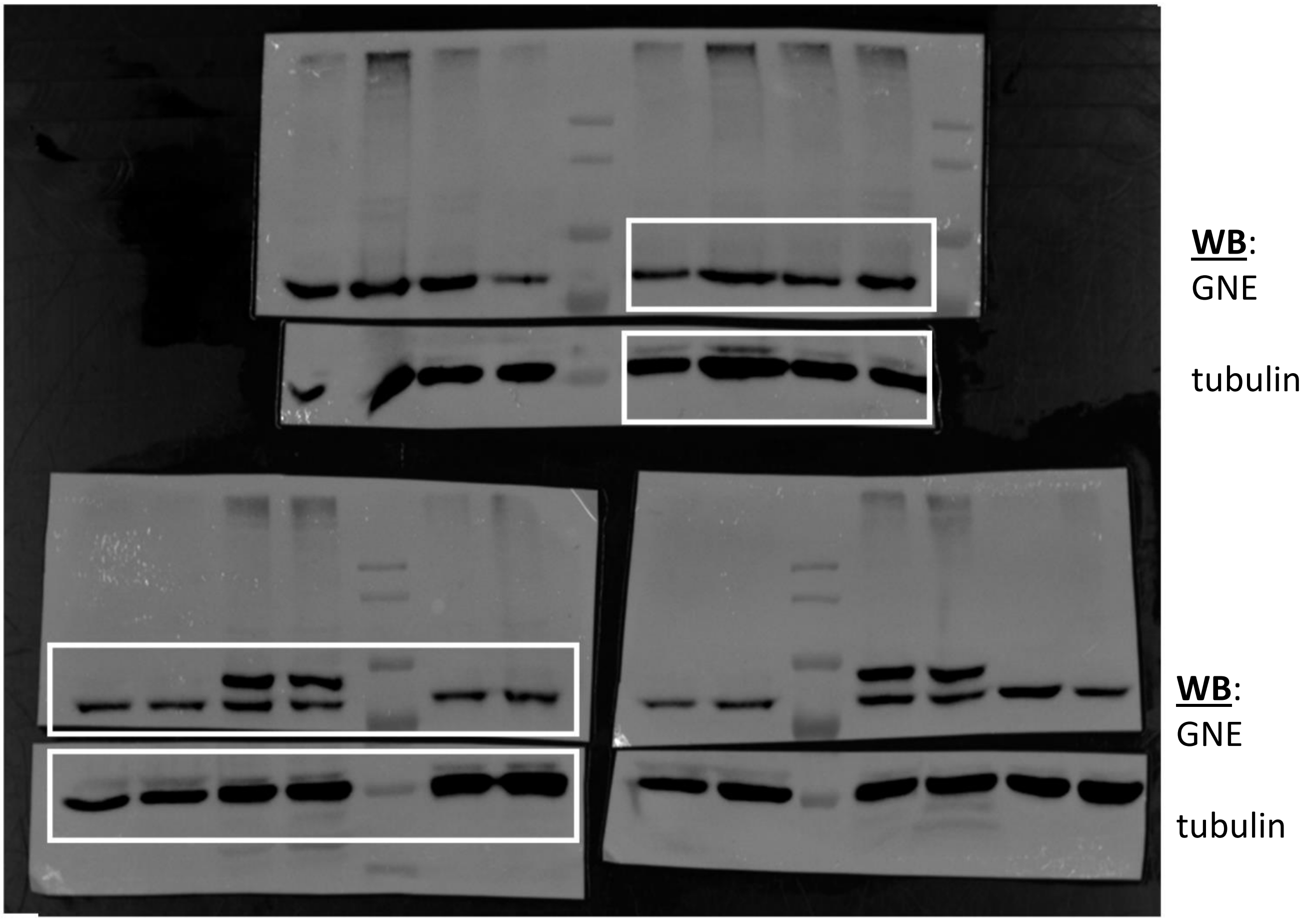

Supplementary Info: Figure S6, uncropped images

WB: Biotin

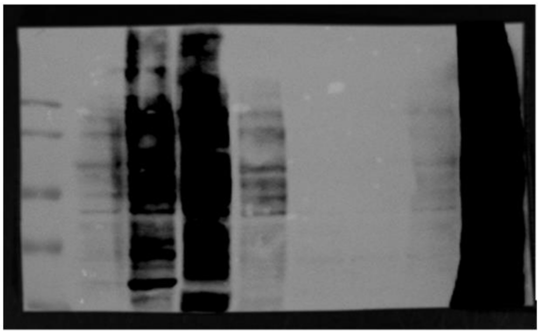

CCD841CoN

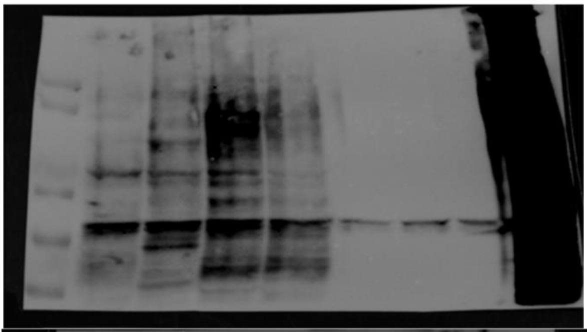

HT29

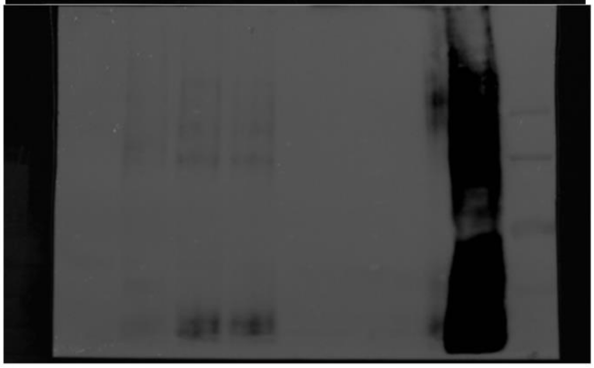

HCT116

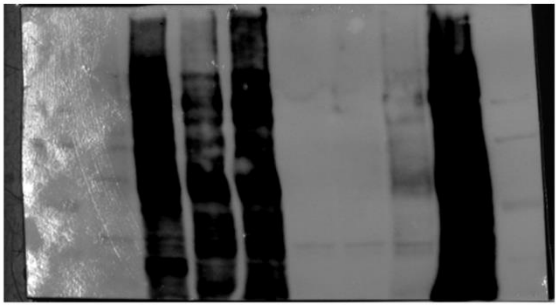

HEK293

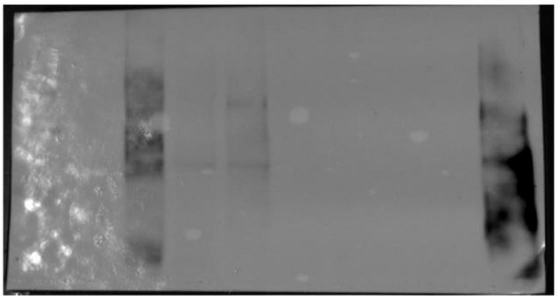

HeLa

Supplementary Info: Figure S7, uncropped images

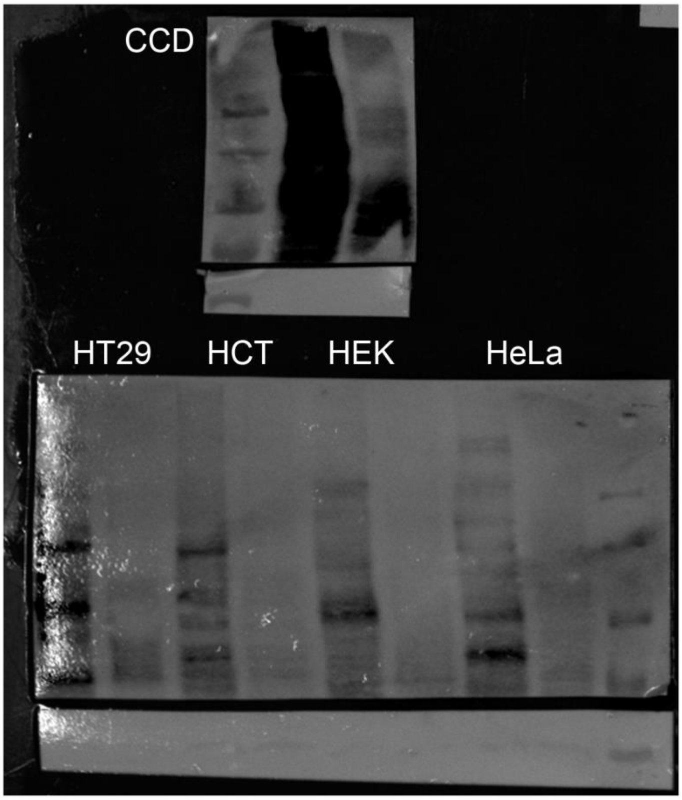

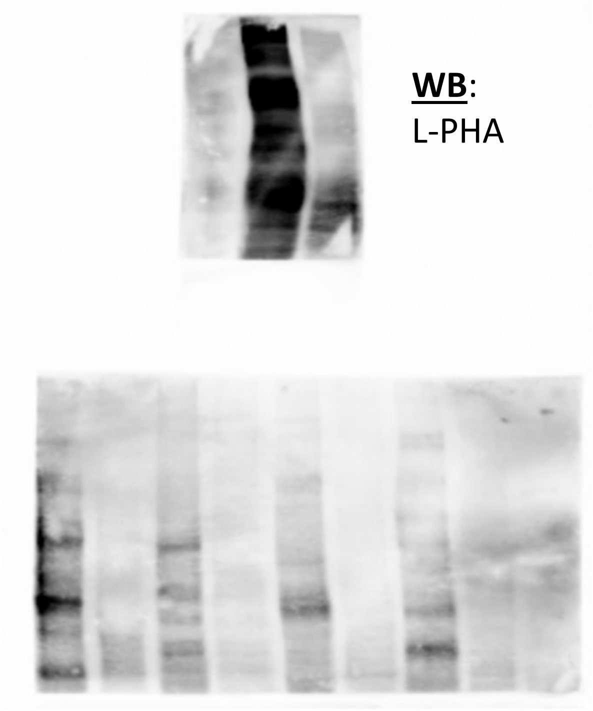

WB:
L-PHA

Supplementary Info: Figure S8, uncropped images

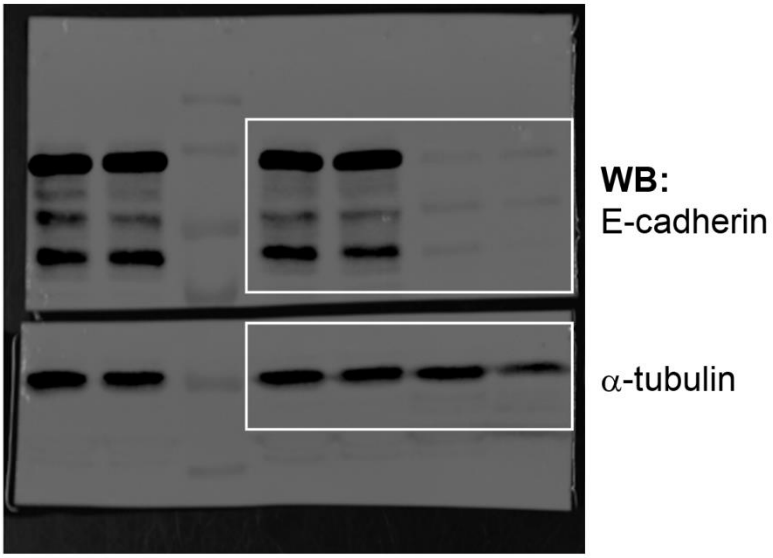

Supplement: Supplementary file 10 — Supplementary Information 10. [file 41598_2022_26521_MOESM10_ESM.pdf]
